# Interleukin‐24: A Multidimensional Therapeutic for Treatment of Human Diseases

**DOI:** 10.1002/wnan.70013

**Published:** 2025-05-07

**Authors:** Rajeswari Raguraman, Anupama Munshi, Rajagopal Ramesh

**Affiliations:** ^1^ Department of Pathology, and OU Health Stephenson Cancer Center University of Oklahoma Health Sciences Oklahoma City Oklahoma USA; ^2^ Department of Radiation Oncology, and OU Health Stephenson Cancer Center University of Oklahoma Health Sciences Oklahoma City Oklahoma USA

**Keywords:** cancer, interleukin‐24, nanomedicine, targeted therapy

## Abstract

The field of targeted therapy exploits the selective expression of therapeutic genes or proteins in diseased cells. While this area is gaining attraction in the context of cardiovascular diseases, diabetes, and other major health disorders, it has been most extensively explored in the realm of cancer. Targeted therapy has gained significance in the cancer field for its potential to address the limitations of conventional treatments and enhance patient survival. Interleukin‐24 (IL‐24), a versatile cytokine, has been evaluated as a cancer therapeutic in various preclinical cancer models and clinical trials, and has yielded promising results. Consequently, multiple studies highlight IL‐24 as a viable “anti‐cancer” therapeutic, with successful outcomes observed in combination therapies involving small molecule inhibitors, chemotherapeutic drugs, and radiation. Despite the evidence validating the tumor‐suppressing properties of IL‐24 in cancer models, there is a dearth of information regarding its role in other human diseases. The objective of this review is to offer a synopsis of the potential role of IL‐24 in diverse human diseases. Additionally, it provides a comprehensive review of the progress in cancer therapy utilizing IL‐24. Finally, from the author's standpoint, the review also addresses some of the limitations that impede the translational potential of IL‐24‐based therapy in clinical settings. It offers arguments in favor of incorporating IL‐24‐based targeted therapy as an effective and safer alternative for current treatment modalities, thereby highlighting its potential to revolutionize the field of therapeutics.

## Graphical abstract

The discovery of interleukin (IL)‐24 and studying its biological activity demonstrate its potential to serve as a therapeutic for human diseases, especially for cancer treatment. Image created with BioRender.com.

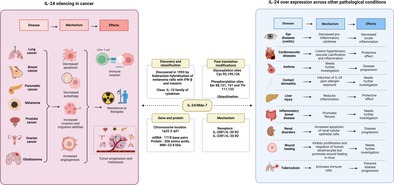

## 1 Introduction

The alarming increase in the global incidence of life‐threatening and life‐limiting diseases has placed an enormous burden on the health care systems worldwide. To address this global healthcare challenge requires extensive efforts to identify and characterize the key signaling components contributing to the development and progression of such diseases. Achieving this goal would help reveal novel genomic targets that can revolutionize targeted therapy and disease management. In the context of disease biology, cytokines, which are signaling molecules released by cells and involved in various inter‐ and intracellular signaling processes, have emerged as key targets for intervention. Specifically, the interleukin (IL)‐10 family of cytokines, comprising IL‐10, IL‐19, IL‐20, IL‐22, IL‐24, IL‐26, as well as the distantly related IL‐28A, IL‐28B, and IL‐29, have garnered significant attention for their potential in treating human disease (Wang et al. 2019). The IL‐10 family of cytokines based on their function can be classified into three groups: (a) IL‐10; (b) the IL‐20 family of cytokines (IL‐19, IL‐20, IL‐22, IL‐24, and IL‐26); and (c) the IL‐28 subfamily (IL‐28A, IL‐28B, and IL‐29) also referred to as type III interferons (IFNs) (Ouyang and O'Garra 2019). These cytokines play a vital role in upholding tissue integrity and homeostasis within epithelial tissues. Additionally, these cytokines mediate immune responses and play a role in the pathogenesis of the central nervous system (CNS), autoimmunity, infection, and inflammation (Burmeister and Marriott 2018; Ouyang et al. 2011). Hence, numerous clinical trials focused on modulating some of the IL‐10‐related cytokines are underway for treating inflammatory and autoimmune diseases (Ouyang and O'Garra 2019). This review focuses on providing an overview of recent progress in understanding the biology of IL‐24 and its downstream signaling and its utilization as a therapeutic tool for cancer management and other human diseases.

## 2 IL‐24

IL‐24, originally referred to as melanoma differentiation associated protein‐7 (mda‐7), was discovered from the human HO‐1 melanoma cell line following treatment with recombinant interferon‐beta (IFN‐β) and mezerin, a protein kinase C (PKC) activator. Introduction and exogenous expression of IL‐24 in human melanoma cell lines halted cell proliferation, demonstrating for the first time its potential antitumor activity (Jiang et al. 1995). Radiation hybrid mapping studies revealed IL‐24 located on chromosome 1 at 1q32.2‐q4. Interestingly, IL‐10, IL‐19, and IL‐20 are also found on this chromosome, albeit at different regions. Another cluster, comprising IL‐22, IL‐26, IL‐28 A, IL‐28 B, and IL‐29, is positioned on a different chromosome. The IL‐24 gene includes seven exons and six introns and encodes a 206 amino acids long protein with a predicted size of 23.8 kDa (Huang et al. 2001). IL‐24 exhibits sequence homology ranging from 15% to 40% with other IL‐10 family members. Additionally, IL‐24 mRNA harbors three AU rich elements at the 3′ untranslated region (UTR), a consensus sequence (AUUUA) and polyadenylation signals (AAUAAA) contributing to the stability and regulation of mRNA, respectively (Madireddi et al. 2000). The presence of three glycosylation sites (cysteine 95, 109, and 126) and five phosphorylation sites (serine 88, 101, and 161, and threonine 111 and 133) in the protein facilitates post‐translational modifications (PTMs) that have been validated both in our laboratory and by others. These PTMs give rise to various isoforms of IL‐24 ranging from 18.3 to 35 kDa in size. Protein expression analysis showed IL‐24 is detected in immune‐associated cell types such as those found in the spleen, peripheral blood mononuclear cells (PBMC), and thymus cells (Huang et al. 2001). Moreover, IL‐24 cDNA contains a secretory signal sequence, facilitating the secretion of the IL‐24 protein. This enables IL‐24 to exert both autocrine and paracrine functions by binding to its two receptor complexes: (a) the IL‐20 heterodimeric receptor complex comprising IL‐20R1/IL‐20R2; and (b) the IL‐22R1/IL‐20R2 complex (Wang et al. 2002). IL‐24 binds to both receptor complexes with equivalent affinity, typically indicated by a dissociation constant (*K*_*d*_) ranging from 2 to 8 nm (*K*_*d*_ = 2–8 nm) (Wang and Liang 2005). IL‐24 binding to its receptor preferentially and greatly activates STAT3 signaling and, to a lesser extent, STAT1 signaling (Chada, Sutton, et al. 2004).

IL‐24 orthologs have been identified in mice and rats. The ortholog, C49a/mob‐5, is a 183 amino acid protein and has a molecular weight ranging from 21.1 to 23 kDa. It is linked with processes involved in wound healing (Soo et al. 1999) and activation of ras oncogenes (Zhang et al. 2000). FISP, the mouse ortholog, selectively expressed in Th2 cells, is a 220 amino acid protein with a relative molecular weight of 25 kDa (Schaefer et al. 2001). The rat and mouse orthologs share ~59% and 69% similarity to human IL‐24, respectively (Schaefer et al. 2001; Soo et al. 1999). However, the biological function of these orthologs differs from IL‐24 and is less extensively studied. This review explores the role of IL‐24 in a spectrum of human diseases, encompassing ophthalmic and cardiovascular disorders, wound healing, inflammation and injury, and infections The later sections of the article provide a detailed exploration of IL‐24 in the context of cancer, discussing the key molecular mechanisms and signaling pathways modulated by IL‐24. We also discuss IL‐24‐based therapies currently in preclinical studies or clinical trials, as well as present our perspective on the prospects for IL‐24‐based therapy in a clinical setting.

### 2.1 Eye Disorders

The exploration of IL‐24's role in ophthalmic disease has not been exhaustively explored with a limited number of studies outlining its involvement in eye disorders. Proteomic studies of human aqueous humor samples showed abundant expression of the IL‐10 protein family, including IL‐20 and IL‐24 (Chowdhury et al. 2010). Moreover, IL‐24 was found to be upregulated in the retinas of DBA/2J mice, which serve as an experimental model of glaucoma characterized by elevated intraocular pressures, age‐related changes, and optic nerve defects (Howell et al. 2011). Likewise, Ross et al., showed that IL‐24 promotes microbial keratitis induced by *Pseudomonas aeruginosa* in the corneas of C57BL/6 mice, while silencing of IL‐24, impacted cytokine expression and reduced the severity of keratitis (Ross et al. 2017). Current research is focused in investigating IL‐24 as a therapeutic for various eye disorders. For instance, intravitreal injection of IL‐24 in an experimental model of autoimmune uveitis decreased ocular inflammation by suppressing differentiation of Th1 and Th17 cells and reduced expression of pro‐inflammatory cytokines (IL‐1β, IL‐6, CCL2, and CCL20) (Zhang et al. 2022). All of these findings while demonstrating a role for IL‐24 in the pathology of ocular diseases, further in‐depth studies are warranted to fully comprehend its function in this context.

### 2.2 Cardiovascular Diseases

Cardiovascular diseases (CVD) affect the heart or blood vessels and are the foremost health challenge worldwide. The etiology of CVDs is complex, with various factors, including gene alterations, metabolic changes, and abnormal protein function linked to their development (Tanai and Frantz 2015). Hypertension, obesity, diabetes, and vascular calcification are all now established risk factors for CVD (Amini et al. 2021). Recently, the involvement of IL‐24 in the biology of CVD has been put forth. For example, specific *IL‐24* gene polymorphisms (rs1150253, rs1150256, rs1150258, and rs3762344) are reported to heighten the risk of CVD (Vargas‐Alarcon et al. 2014).

*Hypertension* is acknowledged as a noteworthy risk factor for CVD. In hypertensive rats, IL‐24 expression was observed to be lower compared with healthy controls; however, it increased following anti‐hypertensive therapy (Wang et al. 2016). Using DNA microarray analysis in spontaneously hypertensive rat models treated with the anti‐hypertensive drugs enalapril and nifedipine, Lee et al. identified 16 genes, including IL‐24, implicated in the pathophysiology of CVD. Treatment of mouse vascular smooth muscle cells with exogenous IL‐24 led to decreased expression of genes encoding products associated with vascular inflammation and hypertension (i.e., angiotensinogen, endothelin‐1, angiotensin receptor‐associated protein, and platelet‐derived growth factor) induced by H_2_O_2_ treatment (Lee et al. 2013). Additionally, recombinant human IL‐24 has been assessed as a therapeutic agent for treating β‐glycerophosphate (β‐GP) induced vascular smooth muscle cell calcification in a rat model.

Vascular calcification that occurs due to mineral deposits in heart valves is commonly associated with the aging process and poses a significant risk for CVD. Treatment with IL‐24, however, inhibited β‐GP‐induced cellular apoptosis, reduced the expression of multiple calcification and osteoblastic markers (BMP‐2, Runx2, ALP, OCN, OPN, MMP‐2, and COLIA1), and suppressed the activation of the Wnt/β‐catenin pathway induced by β‐GP, which is pivotal in vascular calcification (Lee et al. 2012). The outcome of adenovirus‐mediated overexpression of IL‐24 on different rat smooth muscle cells (SMC) has also been investigated since abnormal SMC proliferation and migration are important in proliferative vascular diseases; IL‐24 induced apoptosis and reduced cell viability and migration in pulmonary artery SMC. However, it exhibited no impact on primary human coronary artery SMC or rat aortic SMCs (Chen et al. 2003). In summary, the results from these studies propose a protective effect of IL‐24. However, additional investigation is warranted to comprehensively illuminate the role of IL‐24 in the pathology of CVD.

### 2.3 Allergic Diseases

Allergic conditions, including asthma, contact dermatitis, allergic rhinitis, and urticaria, result from an impaired immune system. They are primarily caused by genetic or environmental factors, including the microbiome, bacteria, viruses, and allergen exposure (chemical, food, or aeroallergens). The incidence of allergic diseases has risen in recent decades, and current treatments predominantly focus on antihistamines, glucocorticosteroids, decongestants, and anti‐leukotrienes, as well as to a lesser extent allergen specific immunotherapy (Wang et al. 2023). Preliminary studies indicate an association between IL‐24 and allergic diseases.

#### 2.3.1 Asthma

Zeissler et al. compared nasal lining secretions and sputum from seasonal allergic asthma patients and healthy controls to identify biomarkers for asthma. IL‐24 displayed moderate correlations as a biomarker linking the upper and lower airways. Furthermore, IL‐24 levels in the sputum were approximately two‐fold higher in seasonal asthma patients (1084.0 ± 199.6 pg/mL) compared with patients out of season (641.2 ± 131.7 pg/mL). Follow‐up analysis utilizing flow cytometry revealed a modest negative association of regulatory T (Treg) cell (characterized by CD25 ^+^/CD127^−^) counts and sputum IL‐24 levels, possibly suggestive of reduced inflammation (Zissler et al. 2018). Another subtype of asthma known as “neutrophilic asthma” is characterized by increased neutrophilic counts in the lungs and airways, which results in more severe asthma with frequent exacerbations that require regular hospitalizations. Feng et al. in their study found elevated expression of IL‐24 and IL‐17A in the airway epithelium of mouse models of neutrophilic asthma and demonstrated that exogenous administration of IL‐37 or siRNA‐based IL‐24 silencing reduced IL‐17A levels and decreased the severity (Feng et al. 2022).

#### 2.3.2 Dermatitis

Investigation into the role of IL‐24 in para‐phenylenediamine (PPD)‐induced allergic contact dermatitis (ACD) showed elevated IL‐24 levels in skin samples from patients allergic to PPD. The IL‐20 family of cytokines, including IL‐24, was shown to be induced in a mouse model of PPD‐induced ACD. Subsequent studies showed that mouse models deficient in IL‐24 were only partially shielded from PPD‐induced ACD (Van Belle et al. 2019). Similarly, elevated IL‐24 and IL‐19 expression has been reported in a mouse model of contact dermatitis triggered by 2,4‐dinitrofluorobenzene exposure. These studies suggest potential connections between IL‐24 and dermatitis, although further research is needed to elucidate these links in detail (Kunz et al. 2006).

#### 2.3.3 Urticaria

Results from auto‐IgEome analyses through microarray technology identified a significant expression of immunoglobulin (Ig) E autoantibodies to IL‐24 in a sizable cohort of chronic spontaneous urticaria (CSU) patients. Further, in vitro functional studies indicated that IL‐24 could trigger the release of histamine in peripheral CD34 ^+^ stem cell‐derived human mast cells sensitized with purified IgE from patients with CSU, yet not in mast cells from healthy controls (Schmetzer et al. 2018). Another study examined *IL24* mRNA levels in skin biopsies from CSU patients and healthy controls; higher levels of *IL24* mRNA were found in spontaneous wheals from CSU patients than in either non‐lesion skin from CSU patients or skin from healthy controls. Additionally, PBMC cultures derived from CSU patients showed higher IL‐24 mRNA levels and protein levels compared with controls. The study also reported a positive correlation between IL‐24 and IL‐6 expression, as well as a weak correlation with disease activity in CSU (de Montjoye et al. 2019).

### 2.4 Inflammation and Injury

IL‐24 has also been investigated in the context of inflammation and injury. In a mouse study, administration of IL‐24 reportedly protected mice against thioacetamide (TAA) by reducing inflammation of the liver through antioxidant effects. Mechanistically, IL‐24 shielded hepatocytes from apoptosis induced by TAA and hindered liver fibrosis by inhibiting the activation of hepatic stellate cells (Wang, Huang, et al. 2021). Numerous investigations have delved into the involvement of IL‐24 in inflammatory bowel disease (IBD). Andoh et al. found elevated IL‐24 mRNA levels in the lesions of patients with ulcerative colitis and Crohn's disease (CD), both types of IBD, compared with normal mucosal samples. Localization studies revealed IL‐24 in sub‐epithelial regions in the mucosal tissues of these patients, and human colonic sub‐epithelial myofibroblast cells were identified as a primary source of IL‐24 in the inflamed mucosa of IBD patients (Andoh et al. 2009). Similarly, colon biopsy specimens from both adult and pediatric patients with IBD have shown positive expression of IL‐24, with documented potential for promoting fibrosis. Similarly, Onody et al. in their study demonstrated elevated IL‐24 expression in serum samples of children diagnosed with IBD as well as in experimental mouse models of dextran sodium sulfate‐induced colitis. IL‐24 treatment of CCD‐18 Co colon fibroblasts in co‐culture models resulted in increased migration and elevated mRNA expression of matrix metalloproteinases (MMPs), tissue inhibitors of metalloproteinases (TIMPs), collagen (COL3A2) and fibronectin (FN1), suggesting a potential role for IL‐24 in promoting colon tissue remodeling (Onody et al. 2021).

Bronchopulmonary dysplasia (BPD) is an inflammatory disease affecting infants and is associated with impaired neurodevelopment, increased pulmonary infections, and frequent hospitalization. Recent evidence suggests that inflammation and oxidative stress are contributing factors in the progression of BPD. Gao et al. identified IL‐24 as a possible target cytokine for BPD. A decrease in IL‐24 production coincided with the fetal alveolar type II cells (FATIIC) Differentiation. Additionally, the administration of IL‐24 increased FATIIC proliferation, while knockdown of IL‐24 using siRNA in the same model system increased expression of SOCS‐3 and caspase‐3, potentially resulting in cell death (Gao et al. 2021).

IL‐24's role in the context of renal disorders has also been investigated. Tabata et al., identified IL‐24 expression as a diagnostic biomarker for predicting the severity of acute kidney injury. Elevated expression of IL‐24 mRNA was observed in kidneys experiencing severe ischemic damage, while elevated IL‐24 levels were detected in both serum and urine in models of ischemic damage. Under hypoxic conditions kidney tubular epithelial cells induced the expression of IL‐24 in vascular SMC (Tabata et al. 2020). A follow up study corroborated these findings and utilized a mouse model to establish upregulated IL‐24 expression, along with upregulation of IL‐24 receptors, following renal‐ischemia reperfusion surgery. Cell culture studies demonstrated that IL‐24 functioned through the endoplasmic reticulum (ER) stress response and resulted in increased apoptosis of renal tubular epithelial cells. Moreover, IL‐24 deficiency, characterized by decreased ER stress and inflammatory markers, was shown to protect mice against ischemia reperfusion injury (Schutte‐Nutgen et al. 2022).

### 2.5 Wound Healing

Investigation into IL‐24 protein expression in normal human skin melanocytes (Ekmekcioglu et al. 2001; Poindexter et al. 2010) suggests its involvement in skin biology. Evidence also indicates IL‐24 receptor expression in keratinocytes (Wang et al. 2002) and the presence of IL‐24 during keratinocyte‐mediated wound repair (Poindexter et al. 2010). Nonetheless, the exact contribution of IL‐24 in wound healing in humans remains unclear and requires further investigation. Stimulation of normal human epidermal keratinocytes with the cytokines involved in wound repair, that is, TGF α and β, IFN β γ induces IL‐24 expression. Additionally, in vitro assays for wound repair and migration illustrated that IL‐24 suppresses TGFα induced proliferation and migration of normal human epidermal keratinocytes (Poindexter et al. 2010).

However, data from mouse models supports the importance of IL‐24 in wound healing. Soo et al. examined murine models with excisional wounds and revealed the expression of c49a (rat ortholog of IL‐24), with immunohistochemistry showing expression primarily localized to fibroblasts present at the wound site (Soo et al. 1999). Subsequently, Kolumam et al. demonstrated high mRNA expression of the IL‐20 family of cytokines, including IL‐24, in skin samples from an experimental mouse splinted wound healing model at day 1, with a peak between day 6 and day 8, thereby suggesting a potential for IL‐24 in accelerating wound healing in mice (Kolumam et al. 2017). IL‐24 and its orthologs could have distinct roles regarding wound healing in mice and humans, thereby requiring further investigation.

### 2.6 Bacterial and Viral Infections

IL‐24 based therapy has also proven effective in the management of infection by intracellular pathogens, especially *Mycobacterium tuberculosis* (Mtb) infection. Higher IL‐24 levels are seen in patients with latent tuberculosis (TB) (Wu, Huang, Kato‐Maeda, et al. 2007), which decrease with TB progression in both humans and Mtb‐infected mice (Ma et al. 2011). Additionally, administration of exogenous IL‐24 in a mouse model infected with the Mtb strain H37Rv effectively protected against Mtb infection. IL‐24, via activating CD8^+^ T cells, stimulated IFN‐γ production and reportedly neutralized Mtb infection (Ma et al. 2011; Wu et al. 2008).

*Mycobacterium bovis* bacille Calmette‐Guérin (BCG) vaccination is administered widely to children for tuberculosis prevention. In a study of neonates administered the BCG vaccine, increased expression of IL‐1, IL‐6, and IL‐24 was observed, contributing to a pro‐inflammatory response and further strengthening evidence of the protective function of IL‐24 in Mtb infection (Wu, Huang, Garcia, et al. 2007).

A similar protective effect of IL‐24 was seen in a mouse model of typhoid fever (*Salmonella typhimurium* C5). This protective effect was reduced upon treatment of the mice with a mutant IL‐24 lacking all three N‐glycosylation sites. IL‐24 induced neutrophils to produce IFN‐γ, NO, and IL‐12, leading to activation of CD8^+^ T cells and a robust host response to combat infection (Ma et al. 2009). IL‐24 production is induced during infection with *Staphylococcus epidermidis* and *S. saprophyticus* (Buzas and Megyeri 2006), and IL‐24 plays an immunosuppressive role during *S. aureus* infection. Elevated levels of IL‐24 were seen in *S.* *aureus‐infected* skin samples. IL‐24, in conjunction with IL‐19 and IL‐20, downregulates IL‐1β and IL‐17A, expediting cutaneous *S. aureus* infection in mouse models (Myles et al. 2013). In conclusion, it can be stated that IL‐24 demonstrates a dual function in immune system response against bacterial and viral infections, with its role being contingent upon the specific infection model being investigated.

### 2.7 Cancer

During cancer progression, there is a notable loss of IL‐24 expression in cancerous cells. Research indicates that while IL‐24 mRNA is detectable, protein expression is absent in both patient tissues and human cancer cell lines. Furthermore, information pertaining to IL‐24 gene mutations or polymorphisms contributing to cancer progression is unavailable. Accumulating evidence from experimental and clinical datasets consistently demonstrates decreased IL‐24 levels in lung (Ramesh et al. 2004), colorectal (Zhang et al. 2019), pancreatic (Jia et al. 2016), endometrial (Liao et al. 2020), head and neck (Qiu et al. 2020), ovarian cancers (Gopalan et al. 2007), melanoma (Ekmekcioglu et al. 2001), and glioblastoma (Lin et al. 2021), with no discernible impact on normal cells. Additionally, in some epithelial cancers such as non‐small cell lung cancer (NSCLC) and melanoma, as well as hematopoietic tumors like diffuse B‐cell lymphoma and Burkitt lymphoma, the absence of IL‐24 protein expression correlates with disease stage and is indicative of a poorer prognosis. Studies have revealed the presence of both IL‐24 mRNA and protein expression in normal melanocytes as well as early‐stage melanomas, which decline with disease progression (Ekmekcioglu et al. 2001), contributing to invasive and metastatic characteristics in melanoma (Ellerhorst et al. 2002). Table 1 summarizes the association between IL‐24 expression and disease pathology across various cancer types. These findings reveal IL‐24 to play a crucial role in suppressing tumor growth, and its loss may contribute to the progression of tumors. Understanding the biology of IL‐24 in relevance to cancer progression has become a focal point of research. In this regard, it has been observed that PTMs are crucial for IL‐24 anti‐cancer activity. While early studies by Sauane et al. concluded that glycosylation is not necessary for IL‐24 anti‐tumor activity (Sauane et al. 2006), our own studies showed that IL‐24 phosphorylation is essential for protein secretion, localization, signaling, and anti‐cancer activity (for instance decreased cell proliferation and invasion as well as induction of cell cycle arrest) (Panneerselvam, Shanker, et al. 2015).

**TABLE 1:** The connection between mda‐7/IL‐24 expression and disease pathology across cancer types.

	Cancer type	Sample size	mda‐7 expression	Correlation with patient disease pathology	References
1	Diffuse large B cell lymphoma (DLBCL)	72 patients with DLBCL and 36 patients without carcinomas	Low expression levels of Mda‐7/IL‐24 (44.44%) were seen in 32 DLBCL tissues	Worse prognosis of DLBCL is predicted by low Mda‐7/IL‐24 expression and high C‐myb expression	Ma et al. (2018)
2	Melanoma	3 melanoma subcutaneous and 3 melanoma lymph node metastasis patients with 3 nevi controls	Loss of mda‐7s in melanoma metastasis patients	The absence of the mda‐7 splice variant contributes to metastatic melanoma	Allen et al. (2004)
3	Melanoma	Melanoma tumor specimens exhibiting primary skin lesions and lymph node metastases	Loss of mda‐7 expression in metastatic melanomas and intense mda‐7 staining in the thinner primary tumors, and very light or no staining in thick primary tumors	Expression of mda‐7 is downregulated in melanomas	Ekmekcioglu et al. (2001)
4	Burkitt lymphoma	59 cases of Burkitt lymphoma patients	Low levels of mda‐7 expression in advanced stage Burkitt lymphoma patients	Poor prognosis of Burkitt lymphoma patients can be predicted by low expression levels of mda‐7/IL‐24 along with high expression of C‐myb	Ma et al. (2018)
5	Breast cancer	127 breast cancer tissues and 31 normal tissues	A significant presence of MDA‐7 within normal mammary epithelial cells contrasts with nearly undetectable staining in cancerous cells	Low mda‐7 levels predict poorer prognosis in breast cancer patients as well as correlate with significantly shorter disease‐free survival	Patani et al. (2010)
6	Non‐small cell lung cancer (NSCLC)	183 patients with resected pathologic stage I‐IIIA, NSCLC	Higher expression of mda‐7 in 97/183 (53.0%) patients	No substantial correlation between MDA‐7/IL‐24 status and patient demographics	Ishikawa et al. (2005)

**FIGURE 1:**
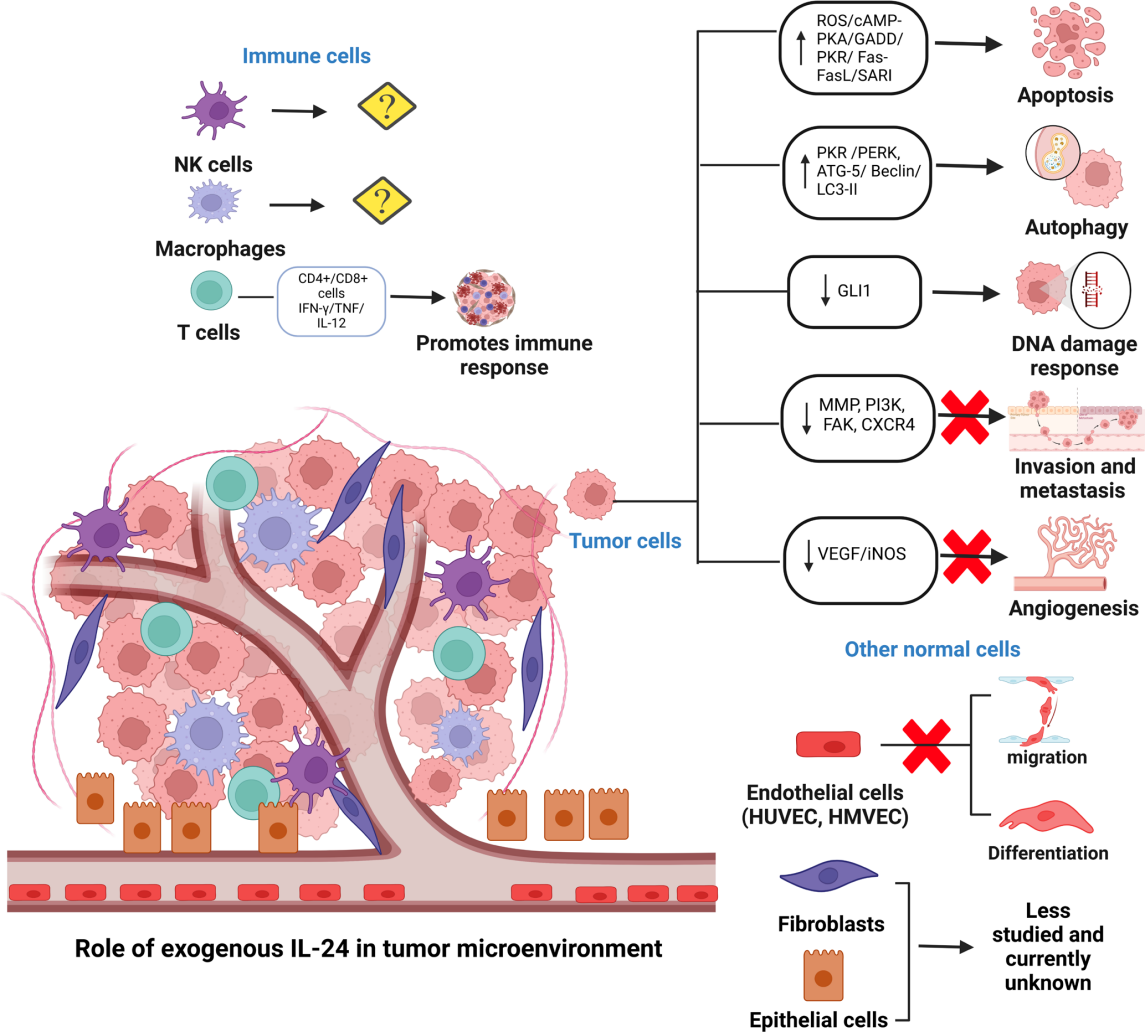
The antitumor activity of exogenous IL‐24 on different cell types in the tumor microenvironment. Reintroduction of exogenous IL‐24 through viral and non‐viral delivery methods in different tumor models demonstrated increased apoptosis, autophagy, DNA damage, and reduced tumor angiogenesis, invasion, and metastasis. Antitumor activity of IL‐24 has also been shown to be mediated in part by stimulating CD4^+^/CD8^+^ T cells and Th1 cytokine release, exerting an immune response. The effect of IL‐24 on other immune cell subtypes (B‐cells, macrophages, natural killer cells), fibroblasts and epithelial cells is limited. This image was created using biorender.com.

The molecular mechanism driving the anti‐cancer activity of IL‐24 seems to vary depending on the specific cancer model under investigation. Nevertheless, critical pathways influenced by IL‐24 encompass MAPK [JNK, ERK], PKR, PERK, Fas/FasL, AKT, beta‐catenin/Wnt signaling, mTOR, STAT‐3, NF‐κB, Gli‐Hedgehog signaling, and iNOS, as extensively reviewed in detail by Panneerselvam et al. and illustrated in Figure 1 (Panneerselvam et al. 2013). The subsequent sections delve into the role of IL‐24 in different facets of tumor progression as well as its utility as an anti‐cancer therapeutic.

#### 2.7.1 IL‐24 Induces Tumor Cell Apoptosis

Apoptosis, also known as programmed cell death, is a pivotal process governing cellular homeostasis. However, cancer cells develop various mechanisms to evade apoptosis, resulting in uncontrolled cellular proliferation and resistance to therapeutic agents, which, in turn, promotes tumor growth and disease recurrence. Thus, a potential strategy to treat cancer would be aimed at employing therapeutic approaches to curb unchecked growth of cancer cells. Two widely adopted approaches for targeting apoptosis in cancer cells involve: (a) stimulating pro‐apoptotic molecules; and (b) inhibiting anti‐apoptotic molecules. In this regard, numerous studies have independently confirmed IL‐24's potential as a potent anti‐cancer agent, capable of selectively triggering apoptosis in various tumor cell types while preserving normal cells. The pathways through which IL‐24 elicits its apoptotic effects have, however, been identified to be cell type dependent.

For example, Chada et al., illustrated that administering adenovirus overexpressing IL‐24 resulted in dose‐ and time‐dependent cytotoxicity in human pancreatic cancer cells (MiaPaCa‐2, AsPc‐1) by regulating Wnt and PI3K pathway proteins (Chada et al. 2005). Similarly, in prostate cancer cells, adenovirus overexpressing IL‐24 induced apoptosis by producing ceramides (C16, C24, and C24:1); myriocin mediated inhibition of serine palmitoyltransferase was found to inhibit ceramide production and thus block IL‐24 mediated apoptosis. Adenovirus overexpressing IL‐24 also increased the expression of acid sphingomyelinase (ASMase), an enzyme catalyzing the hydrolysis of sphingomyelin into ceramide, resulting in increased ASMase activity and reduced sphingomyelin levels in cancer cells. Subsequently, knockdown of ASMase using RNA interference abolished IL‐24 mediated formation of ceramides and abrogated the decrease in cell viability. The study further showed that IL‐24 activated protein phosphatase 2A (PP2A), which subsequently dephosphorylated Bcl2 (Sauane et al. 2010).

IL‐24 also triggers apoptotic responses through endoplasmic reticulum (ER) stress. Its antagonistic effect on Sigma 1 Receptor (S1R) was identified and found to contribute to the induction of apoptosis in prostate cancer models. Adenovirus‐mediated IL‐24 overexpression induced apoptosis in cancer cells by triggering ER stress, characterized by increased p‐eIF2α, CHOP, and BiP and generation of reactive oxygen species (ROS). Subsequently, this led to the activation of caspase‐3, further contributing to cellular apoptosis in prostate cancer cells (Do et al. 2013).

Studies also show that IL‐24 treatment triggers phosphorylation of eiF2α on the serine 51 residue in prostate cancer (Sauane et al. 2008), melanoma, breast, and cervical cancer cells (Persaud et al. 2017). IL‐24 mediated eiF2α phosphorylation is considered pivotal as it induces a global halt in protein translation, consequently decreasing the expression of anti‐apoptotic proteins Mcl‐1 and Bcl‐xL. In breast cancer cells, Persuad et al., demonstrated that the activation of cyclic adenosine monophosphate (cAMP)‐dependent protein kinase A (PKA) precedes apoptotic tumor cell death induced by IL‐24. Inhibiting PKA activity using H‐89 and PKI peptide reversed the IL‐24‐induced apoptotic effects on a panel of breast cancer cells. PKA stimulated phosphorylation of p38 MAPK at threonine 180 and tyrosine 182 residues in MCF‐7 breast cancer cells, leading to the upregulation of Fas, FasL, DR4, and FADD levels, all indicative of an activated extrinsic apoptotic pathway following IL‐24 treatment (Persaud et al. 2018). Mitochondrial dysfunction and the generation of ROS have also been recognized as mediators of IL‐24 induced cellular apoptosis. ROS play a dual role in cancer cell metabolism. Lower levels of ROS stimulate cellular proliferation, whereas higher levels are associated with cell damage and induce apoptosis (Nakamura and Takada 2021). Lebedeva et al. identified the potential link between ROS production and apoptosis in prostate cancer cell models. The use of antioxidants (Tiron and *N*‐acetyl‐l‐cysteine) and chemicals inhibiting mitochondrial permeability (cyclosporine A and bongkrekic acid) when analyzed synergistically with adenovirus‐overexpressed IL‐24 on DU‐145, PC‐3, and LNCaP cells mitigated the cell‐killing effect of IL‐24. Conversely, the use of ROS‐generating agents such as arsenic trioxide (As_2_O_3_), NSC656240 (dithiophene with applications in cancer), and PK11195 (isoquinoline carboxamide) enhanced the IL‐24‐mediated apoptosis (Lebedeva et al. 2003). Adenovirus‐overexpressing IL‐24 prompted apoptosis and induced G2/M cell cycle arrest in melanoma cells, while showing no such impact on normal human melanocytes (Ekmekcioglu et al. 2001). Further investigation revealed that melanoma cells increased the expression of GADD family members—GADD153, GADD34, and GADD45α—in response to IL‐24. Given the involvement of the GADD gene family in the induction of the p38 MAPK pathway, cells were subjected to treatment with the p38 MAPK inhibitor, SB203580, to assess potential effects. Treatment with SB203580 inhibited the expression of genes in the GADD family. Pharmacological (SB203580) and AdCMV‐Flag p38 (AGF)‐mediated p38 MAPK inhibition prevented the downregulation of Bcl2, thereby reversing IL‐24 mediated cellular apoptosis in melanoma cells (Sarkar et al. 2002).

The NF‐κB pathway plays a crucial role in regulating cell survival in tumor cells. The selective tumor‐killing ability of IL‐24 is further substantiated by its ability to suppress NF‐κB expression in human lung tumor cells H1299 and A549. Oida et al. assayed the time‐ and dose‐dependent induction of IL‐24 in relation to NF‐κB expression. Additionally, a H1299 model overexpressing dominant‐negative I kappa B alpha (dnI kappa B alpha) was utilized and exposed to varying concentrations of adenovirus overexpressing IL‐24 and demonstrated enhanced growth arrest and induction of apoptosis through the cleavage of MEKK1 and caspase‐3 in response to IL‐24. Additionally, in vivo studies demonstrated that the same treatment of subcutaneously implanted H1299‐dn I kappa B alpha cells resulted in enhanced drug sensitivity and a hindered tumor growth (Oida et al. 2007).

Another mechanism of IL‐24‐mediated apoptosis documented in lung cancer models stems from upregulation/activation of the double‐stranded RNA‐dependent protein kinase (PKR), which leads to the downstream phosphorylation of eiF2α. Treating cells with 2‐aminopurine (2‐AP) repressed the activation of PKR and IL‐24‐mediated induction of apoptosis (Pataer et al. 2002). A study by Pataer et al. later proposed a physical interaction between IL‐24 and PKR, thereby identifying a mechanism by which IL‐24 post‐transcriptionally regulates PKR and contributes to apoptosis (Pataer et al. 2005). In another study, adenovirus‐mediated IL‐24 overexpression in subcutaneous tumors generated using A549 and H1299 lung cancer cells resulted in significant tumor growth inhibition (40% in A549 and 27% in H1299) through the induction of apoptosis, via modulating CD31/PECAM expression and upregulating APO2/TRAIL (Saeki et al. 2002). Microarray analyses of breast and NSCLC cell lines overexpressing IL‐24 had previously revealed upregulation in the expression of genes with tumor‐suppressing abilities such as E‐cadherin, PTEN, and GSK‐3β. Simultaneously, there was downregulation of genes associated with the PI3K/β‐catenin signaling pathway and a notable shift in β‐catenin levels from the nucleus to the plasma membrane. Interestingly, inhibition of PI3K using the pharmacological inhibitor wortmannin did not negate the cell killing effects of IL‐24. These studies showed that IL‐24 overexpression increased E‐cadherin expression, subsequently inhibiting cellular migration and enhancing cellular adhesion (Mhashilkar et al. 2003). Similarly, treatment of ovarian cancer xenograft models resulted in specific induction of cell cycle arrest and apoptosis, with molecular analyses revealing elevated expression of caspases in tissue lysates from treated tumors (Gopalan et al. 2007). IL‐24‐mediated apoptotic cell death in ovarian cancer cells was found to be a consequence of the activation of transcription factors c‐Jun and ATF2, which, in turn, stimulated Fas/FasL signaling, ultimately converging on NF‐κB and promoting cellular apoptosis. To confirm Fas signaling involvement in IL‐24‐mediated cellular apoptosis, the study utilized siRNA‐mediated Fas inhibition and therapeutic antibody (NOK‐1) based blocking of Fas (Gopalan et al. 2005).

Similarly, researchers have investigated the involvement of c‐Jun N terminal kinase in IL‐24‐mediated cellular apoptosis. The involvement of JNK in modulating both extrinsic apoptotic and intrinsic mitochondrial apoptotic pathways has been extensively reviewed by Dhanasekaran and Reddy (2008). Yacoub et al. observed that radiation enhances the anti‐proliferative activity induced by IL‐24, while overexpressing IL‐24 alone led to an increase in p38 and ERK1/2 activity without affecting JNK expression. Adding radiation resulted in decreased ERK1/2 along with an increase in JNK1/2 levels, with no additional effect on p38 and Akt levels (Yacoub, Mitchell, Lebedeva, et al. 2003). An additional follow‐up study from the same research group investigated the effects of a GST fusion protein containing IL‐24 in glioblastoma models. Data from this study indicated that the collective inhibition of both ERK1/2 and AKT function was essential to elicit cell‐killing effects of GST‐IL‐24. This combined inhibition strategy was found to modulate the JNK1‐3 signaling pathway, resulting in pronounced cell death in primary human glioma cells. Activated JNK1‐3 signaling led to the activation of BAX, resulting in mitochondrial dysfunction to ultimately cause cellular apoptosis (Yacoub et al. 2008).

Adenovirus mediated IL‐24 transduction also led to the induction of suppressor of AP‐1(SARI), induced by IFN expression in multiple cancer models. This induction of SARI was specific to tumor cells and absent in normal cells. A *SARI* ‐antisense‐based approach was further employed to prove the pivotal role of *SARI* in IL‐24‐mediated anti‐tumor effects (Dash et al. 2014).

A recent study conducted by Babazedeh et al. revealed that IL‐24 transduction in glioblastoma cells resulted in reduced cell proliferation. This was achieved via inducing sub‐G1 cell cycle arrest and cellular apoptosis. More importantly, adenovirus‐IL24 transfection increased caspase‐3, TNF‐α, TRAIL, and p38 MAPK levels in U87 cells compared with untreated controls (Babazadeh et al. 2023). These examples illustrate the various mechanisms by which IL‐24 regulates apoptosis in different cancer models. It is important to note that this review on IL‐24‐mediated cancer cell apoptosis is not comprehensive. Readers seeking more depth can explore Emdad et al. (2020) review for additional insights.

#### 2.7.2 Autophagy

Autophagy is an essential component of the cellular self‐degradation process important for maintaining cellular metabolism and homeostasis (Yun and Lee 2018). A cluster of proteins tightly governs the process of autophagy. The autophagic cascade is initiated by the translocation of unc‐51‐like kinase 1 (ULK1) to the phagophore initiation site, triggering its activation. Once the ULK1 complex is activated, it recruits the class III phosphatidylinositol 3‐kinase, composed of beclin‐1, VPS 15, VPS 34, and ATG14L. The formation of this complex triggers the synthesis of PI3‐P, a crucial component of phagophores. PI3‐P, in turn, recruits the autophagy machinery and facilitates the maturation of autophagosomes (Chen et al. 2021; Debnath et al. 2023). Paradoxical roles for autophagy have been recognized in the context of cancer, with documented instances of both promoting and inhibiting tumor growth (Lim et al. 2021). Early studies by Gupta et al. revealed an interaction between IL‐24 and BiP/GRP78 proteins, resulting in the activation of p38 MAPK and the GADD family of genes (Gupta et al. 2006). These preliminary findings concerning IL‐24‐mediated modulation of ER stress along with the UPR/BiP pathway sparked curiosity among researchers, prompting further investigation into IL‐24‐mediated autophagy in cancer cells. Park et al. demonstrated that GST‐IL‐24 induced autophagy in glioma cells by activating JNK1‐3, BAX, and inducing mitochondrial dysfunction. Park et al. determined that GST‐IL‐24 caused glioma cells to undergo autophagy by activating JNK1‐3, BAX, and inducing mitochondrial dysfunction. Another key finding observed in the study was the dependency on protein kinase R‐like endoplasmic reticulum kinase (PERK) for GST‐IL‐24‐mediated activation of JNK1‐3, as evidenced by no significant lethality in PERK ^−/−^ cells. Treatment with GST‐IL‐24 led to PERK‐dependent vacuolization of LC3‐expressing endosomes, which was inhibited by autophagy inhibitors such as 3‐methyladenine, and modulation of proteins such as HSP70, BiP/GRP78, ATG5, or Beclin 5. The authors of this study further established that GST‐IL‐24, through the induction of autophagy, activates various pro‐apoptotic pathways, leading to a decrease in glioma cell survival (Park et al. 2008). Yacoub et al., in their study, revealed that overexpression of IL‐24 activated PKR‐PERK, subsequently triggering the production of ceramides and ROS to elicit autophagic cell death in glioma cells (Yacoub et al. 2010). These findings on IL‐24 involvement in autophagy were further substantiated using OSU‐03012 in glioblastoma models. OSU‐03012 enhanced the IL‐24‐mediated cytotoxicity in GBM cell lines; the combination increased PKR‐PERK while reducing the expression of the anti‐apoptotic proteins MCL‐1 and BCL‐XL, resulting in a profound anti‐tumor effect (Hamed et al. 2010). In a study on renal cell carcinoma, histone deacetylase (HDAC) inhibitors in combination with IL‐24 induced autophagy by modulating the expression of ceramide synthase (Hamed, Das, et al. 2013). Similarly, IL‐24 has been shown to induce ER stress and ceramide production in prostate cancer (Bhutia et al. 2010) and leukemia cells (Yang et al. 2010), leading to an increased expression of autophagy proteins such as beclin‐1, ATG‐5, and hVps 34. Moreover, in U87 glioblastoma cells, in addition to apoptosis induced by IL24 an elevated expression of LC3‐II protein, suggestive of autophagy activation, was also observed (Babazadeh et al. 2023). These examples underscore the pivotal role of IL‐24 in facilitating autophagy‐induced cell death.

#### 2.7.3 IL‐24 Decreases Tumor Invasion and Migration

**FIGURE 2:**
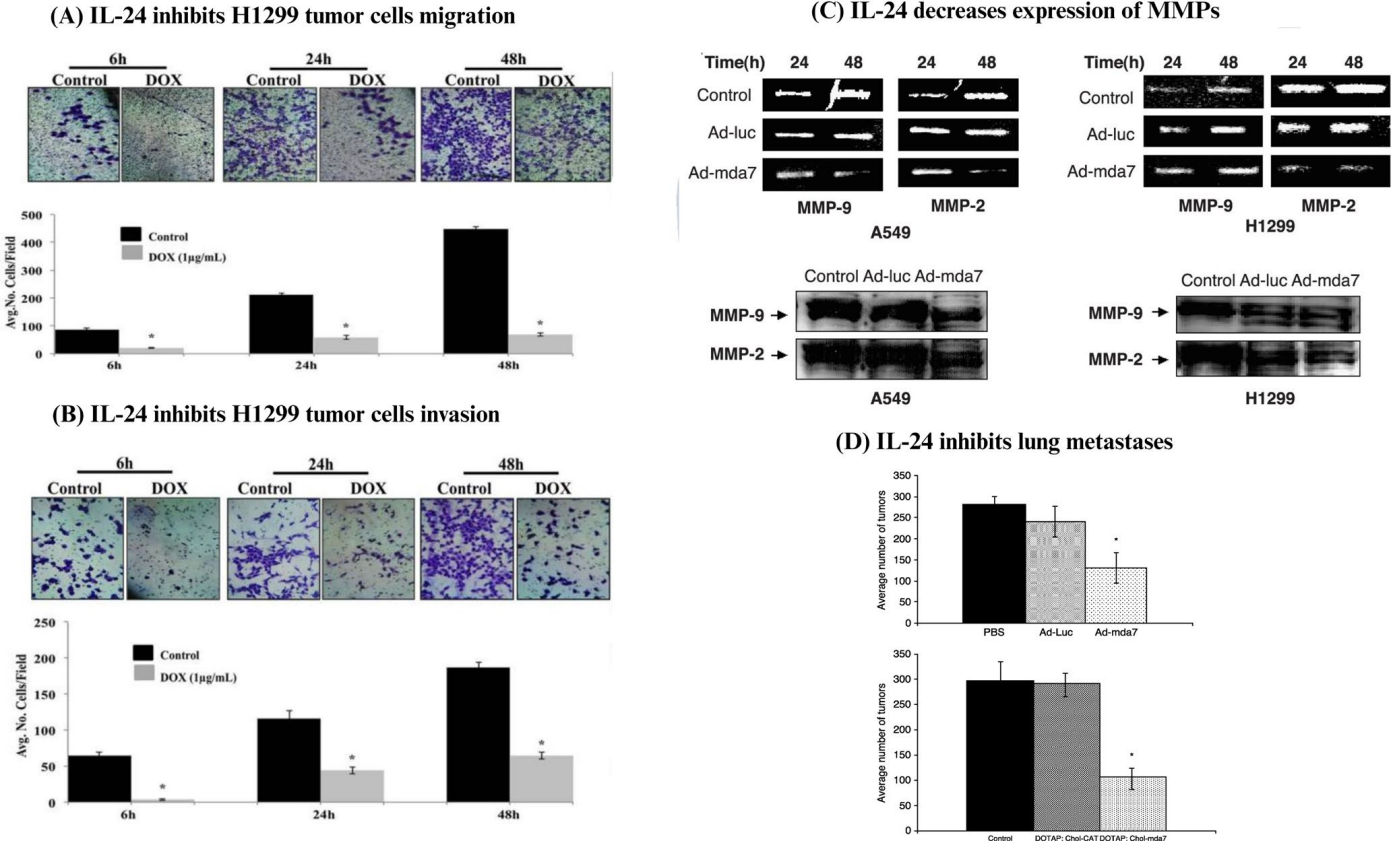
Effect of exogenous IL‐24 on tumor invasion and metastasis. Published reports from our laboratory show that the introduction of IL‐24 via viral and non‐viral delivery systems decreases (A) migratory and (B) invasive abilities of H1299 lung cancer cells and (C) expression of MMPs—MMP‐2 and MMP‐9. Decreased invasive abilities along with a reduction in MMPs further led to (D) reduction in tumor growth in experimental A549 lung metastases models of IL‐24 treated tumors. Images sourced from Panneerselvam, Jin, et al. (2015) and Ramesh et al. (2004) are available under the open access Creative Commons CC BY 4.0 license.

Acquired mutations in cancer cells often confer them with invasive and migratory capabilities, enabling them to enter the lymphatic system and blood vessels for dissemination into the systemic circulation, leading to metastasis at different sites, ultimately giving rise secondary tumors. Factors such as chromosomal instability, gene alterations, intra‐tumoral heterogeneity and the process of epithelial–mesenchymal transition (EMT), have all been linked with promoting migration and invasion, thereby facilitating metastasis (Novikov et al. 2021). In addition to its involvement in cancer cell death, IL‐24 also hampers cell migration and invasion through the downregulation of crucial factors such as MMPs, specifically MMP‐2 and MMP‐9, PI3K, FAK, and the CXCR4 chemokine. Ramesh et al., were among the first to demonstrate IL‐24's ability to impede the migratory and invasive tendencies of lung cancer cells. Preliminary studies in lung cancer revealed that IL‐24 effectively curtailed lung cancer migration and invasion by modulating the expression of PI3K, MAPK and FAK proteins (Figure 2A,B). Additionally, gelatin zymography experiments demonstrated downregulation of MMP‐2 and MMP‐9 expression following IL‐24 induction in H1299 and A549 cells (Ramesh et al. 2004) (Figure 2C). Notably, MAPK and FAK have been well implicated in migration of cancer cells (Hauck et al. 2002; Huang et al. 2004). Similarly, MMPs play a critical role in degrading the extracellular matrix (ECM) components and the basement membrane, facilitating tumor cell invasion into the bloodstream and other tissues, ultimately leading to metastasis. Additionally, MMPs also aid in the reorganization of the ECM facilitating tumor cell migration, and as a result elevated MMP expression is a characteristic feature of cancer. The involvement of MMPs in cancer cell migration, invasion and metastasis has been extensively reviewed by Nabeshima et al. (2002). Moreover Ramesh et al., observed a decrease in metastasis in an A549 experimental lung metastasis model following overexpression of IL‐24 treatment (Figure 2D) (Ramesh et al. 2004).

This decrease in metastasis is possibly a consequence of the diminished abilities of tumor cells to migrate and invade after IL‐24 treatment. Subsequent studies have reported similar findings, showing that IL‐24 treatment inhibits neuroblastoma (Zhuo et al. 2013) and osteosarcoma cell migration (Zhuo et al. 2017) through the modulation of PI3K, FAK, JNK and the inhibition of MMPs. Likewise, other studies have documented that the administration of IL‐24 results in decreased invasive and migratory capacity in Ishikawa cells (Liao et al. 2020) as well as MDA‐MB‐231 breast cancer cells (Patani et al. 2010). These results align with our prior findings and support that IL‐24 plays a crucial role in restraining cancer cell invasion and migration across diverse cancer models. SDF‐1/CXCR4 is another major signaling pathway known to be involved in tumor cell proliferation, migration, and metastasis. Recognizing the critical role of SDF‐1/CXCR4 axis, our laboratory conducted studies to examine the impact of IL‐24 overexpression on key target molecules in the SDF‐1/CXCR4 pathway in lung cancer models. Results identified that IL‐24, via posttranscriptional regulation, caused a decrease in both CXCR4 mRNA and total CXCR4 protein expression. This decrease in CXCR4 levels was achieved by influencing downstream signaling through the AKT/mTOR signaling pathway (specifically pAKT^S473^, p‐mTOR^S2448^, pPRAS40^T246^, and HIF‐1α), which subsequently contributed to decrease in lung cancer cell migration and invasion (Panneerselvam, Jin, et al. 2015). Moreover, our research also demonstrated that IL‐24 overexpression, in combination with knockdown using siRNA of HMGA1, led to significant decrease in Akt levels and impaired invasion and migration of H1299 cells, in comparison to individual monotherapy (Panneerselvam et al. 2016). Moreover, there are reports indicating that IL‐24 is involved in modulating EMT, a process known to promote tumor cell invasion and confer resistance to anti‐cancer therapies. Retroviral‐mediated transduction of IL‐24 in DU‐145 cells, resulted in the upregulation of epithelial markers (E‐cadherin) and a decrease in the expression of mesenchymal markers (fibronectin, α‐SMA, and smad7), providing additional evidence for reduced tumor cell aggressiveness following IL‐24 treatment. Additionally, a decrease in actin fiber formation was documented, suggesting suppressed tumor cell motility (Maehana et al. 2019). Similarly, Goerlich et al., conducted studies in lung cancer cells to evaluate the potential correlation between IL‐24 transduction and the regulation of EMT markers. In a doxycycline‐inducible H1299 stable system, IL‐24 transfection led to decrease in N‐cadherin and TWIST levels, as well as decrease in the mRNA levels of various transcription factors, including Snail, Slug, and Twist. Furthermore, an increase in Zeb/AREB levels was noted in H1299‐IL24 cells compared with controls. These findings propose that IL‐24's modulation of transcription factors involved in EMT might play a role in limiting metastasis (Corbin Goerlich et al. 2010).

#### 2.7.4 Bystander Effect

Earlier sections of this review article explored the secretory signal sequence within IL‐24, which enables it to be cleaved and secreted. While numerous studies have revealed novel pathways modulated by intracellular IL‐24, some efforts have also aimed to explore the impact of secreted IL‐24's anti‐tumor activity on neighboring cells. These studies centered on discovering the possible role of secreted IL‐24, led to the identification of the bystander effect of IL‐24 in cancer models, with the initial documentation of MDA‐7/IL‐24's—bystander effect—occurring in pancreatic cancer cells. While in most cancer cells transfection using replication‐incompetent adenovirus overexpressing IL‐24 led to expression of IL‐24, prostate cancer cells remained an exception. These cells were identified to have a resistant phenotype that impaired their ability to translate IL‐24 mRNA expression into protein. This resistance was attributed to the high occurrence of K‐Ras mutations (85%–95%) in pancreatic cancer cells. To overcome this resistance, anti‐sense approaches were used to downregulate K‐Ras expression in MiaPaCa 2 cells, when combined with transfection of adenovirus overexpressing IL‐24, there was resulting tumor growth suppression, induction of apoptosis in athymic mouse models, and increased production of IL‐24 protein. It was further documented that IL‐24 secreted from a minor subset of tumor cells was able to infiltrate the whole tumor, thus establishing the bystander effect of IL‐24 (Su et al. 2001). Subsequent investigations by Su et al., explored the impact of IL‐24 secretion by normal cells in contributing to this bystander effect. Several normal cell lines, including FM516‐SV (SV40‐immortalized normal melanocytes), P69 (SV40‐immortalized normal prostate epithelial cells) and primary human fetal astrocytes (PHFA)‐IM (hTERT‐immortalized PHFAs) were infected with adenovirus overexpressing IL‐24. A higher level of IL‐24 was reported in PHFA‐IM cells than either FM516‐SV or P69 cells. Subsequently, the ability of IL‐24 secreted from normal cells to exert biological effect on uninfected DU145 cancer cells was evaluated. The findings revealed that IL‐24 secreted from normal cells suppressed anchorage‐independent growth and invasion capabilities of DU145 cells (Su et al. 2005), suggesting that IL‐24 expression in normal cells can influence neighboring cancer cells and underscoring the importance of the bystander effect for IL‐24's potential as an anti‐tumor agent.

Chada et al. assessed the functionality of glycosylated secreted IL‐24 in melanoma cell lines. They administered recombinant IL‐24 protein, which functioned through its type 1 and type 2 IL‐20 receptors, inducing phosphorylation and subsequent nuclear translocation of STAT3. This in turn resulted in a dose‐dependent cell death effect in MeWo cells (Chada, Mhashilkar, et al. 2004). Similar results were also documented in pancreatic cancer, where the addition of exogenous IL‐24 activated STAT3, contributing to tumor cell death. It was noted that the effect of IL‐24 could be neutralized by employing anti‐IL‐24 antibodies or antibodies targeting the IL‐24 receptor, halting the observed cell‐killing (Chada et al. 2005). Similarly, IL‐24 secreted from P69 (normal prostate epithelial cells), when administered to the cancer cell lines DU‐145, BxPC‐3, led to the repression of colony forming abilities and invasiveness. However, no effect was observed on A549 lung tumor cells that lacked IL‐24 receptors (Su et al. 2005). These studies collectively reinforce the idea that the cytotoxicity of IL‐24 is specific to cells containing IL‐24 receptors, while cells devoid of IL‐24 receptors do not exhibit this effect.

In vivo studies were conducted in subcutaneous xenograft tumor models established using a 1:1 ratio of human lung A549 cells with parental HEK293 cells or IL‐24 expressing HEK 293 cells. Animals receiving the mixture of A549 cells and HEK 293 cells expressing IL‐24 exhibited a reduction in tumor growth. ELISA revealed circulating IL‐24 protein in mice serum, and this secreted IL‐24 was reported to affect tumor endothelial cells and hinder angiogenesis, resulting in an anti‐tumor effect (Ramesh et al. 2003). Additional investigation also confirmed the bystander effect of IL‐24 in various cancer types. For instance, Sarkar et al. demonstrated the complete eradication of tumors, both at the primary as well as distant sites, following injection of conditionally replication‐competent adenovirus containing mda‐7/IL‐24 (Ad.PEG‐E1A‐*mda*‐7) into athymic nude mice bearing human breast cancer xenografts (Sarkar et al. 2005). Pradhan et al. conducted a study wherein they demonstrated a more potent bystander effect of Ad5‐M7S (a genetically engineered IL‐24 cytokine with enhanced stability and secretion capability) compared with wild‐type adenovirus carrying IL‐24 in melanoma and prostate cancer xenograft models (Pradhan et al. 2022). This study's findings are elaborated on in greater detail in subsequent sections of this review article. Finally, in a clinical setting involving intra‐tumoral administration of adenovirus expressing IL24 (INGN‐241) to cancer patients, greater tumor cell apoptosis was observed at the tumor site and distant sites, underscoring the pronounced bystander effect of IL‐24 in humans (Cunningham et al. 2005; Tong et al. 2005).

#### 2.7.5 DNA Damage Response

Both exogenous factors (exposure to radiation, chemotherapeutic drugs, chemical pollutants, and other harmful agents) and endogenous processes (increased ROS within a cell) can lead to DNA damage, which manifests as single‐stranded breaks (SSBs) and double‐stranded breaks (DSBs). Multiple DNA repair pathways have been identified, which include the mismatch repair (MMR), base excision repair (BER), and nucleotide excision repair (NER) for repairing SSBs, as well as homologous recombination (HR) and non‐homologous end joining (NHEJ) pathways to repair DSBs. Due to their inherent cellular mechanisms, cancer cells frequently display high levels of DNA damage, making the inhibition of DNA damage response an attractive therapeutic option for cancer management. Several agents targeting the DNA damage response, such as PARP inhibitors, topoisomerase inhibitors, and checkpoint inhibitors, have been used clinically and are extensively reviewed by O'Connor (2015) and Alhmoud et al. (2020). Multiple reports suggest that the expression of GLI1 is linked to DNA damage and contributes to the carcinogenic process (Mazumdar et al. 2011; Meng et al. 2015; Palle et al. 2015). Our group explored the potential of IL‐24 to modulate components of the DDR pathway and showed that IL‐24 selectively inhibited GLI1 expression in NSCLC cells (H1299 and A549) but not in normal cells. IL‐24 was found to post‐transcriptionally regulate GLI1, leading to a noticeable change in the ataxia‐telangiectasia‐mutated (ATM) pathway markers (decrease in pATM, pCHK2, RAD50, and MRE11) which resulted in aberrant DNA damage demonstrated by comet assay and γ‐H2AX foci. Additionally, IL‐24‐mediated suppression of GLI1 correlated with increased cellular apoptosis, characterized by a significant decrease in Bcl2 and cyclin D1 expression, and an increase in cleaved PARP and caspase‐3 levels. The involvement of GLI1 in cellular apoptosis and DNA damage was also examined in the presence of GANT61, a hedgehog inhibitor; H1299‐IL24 cells co‐treated with GANT61 exhibited reduced GLI1 and Bcl2 levels, along with increased γ‐H2AX and cleaved PARP levels (Panneerselvam et al. 2019). These findings support the possibility of IL‐24 playing a role in DDR, which could contribute to promoting tumor cell death.

#### 2.7.6 IL‐24 and the Immune Response

Information pertaining to the immunoregulatory role of IL‐24 is limited in the context of cancer. In vitro studies, using colorectal adenocarcinoma models, have revealed that the effect of IL‐24 stimulation on T cell population depends on the dosage. While lower levels of IL‐24 (10 ng/mL) did not significantly affect T cells, higher concentrations (100 ng/mL) promoted CD4^+^ and CD8^+^ T cell activity and function. This is characterized by elevated percentage of Th1 and Th17 cell populations, enhanced expression of CD4^+^ transcription factors such as T‐bet/RORgt mRNA, IFN‐γ secretion, and diminished frequency of Treg cells, as along with a reduction in FoxP3 mRNA levels and secretion of IL‐10 (characterized by increased perforin, granzyme levels, and induction of cytolytic and non‐cytolytic activity) (Zhang et al. 2019). Ma et al., investigated the role of IL‐24 in suppressing tumor growth using in vivo colon cancer models with an intact immune system. In their study, 5 × 10^5^ CT26 cells were injected subcutaneously followed by treatment with intravenous GST‐IL‐24 fusion protein to examine the effect of IL‐24 on immune cells and tumor growth. IL‐24 was found to enhance CD4^+^ T cells and CD8^+^ T cells ability to produce IFN‐γ, thereby increasing the cytotoxicity of CD8^+^ T cells. Additionally, administration of IL‐24 reconfigured the TME (tumor microenvironment) in murine colon cancer models, with a higher percentage of CD45^+^ CD4^+^ and CD45^+^ CD8^+^ cells and a decrease in CD45^+^ CD4^+^ Foxp3^+^ cells, suggestive of a type I immune response. Furthermore, GST‐IL‐24‐treated mice exhibited a higher number of IFN‐γ and granzyme B‐producing CD8^+^ T cells, as well as IFN‐γ‐producing CD^+^ T cells in comparison to the mice in the control group (Ma et al. 2016). Miyahara et al., also investigated how IL‐24 influences the immune response in a murine syngeneic tumor model. UV2237m cells transduced with adenovirus overexpressing IL24 were injected into both syngeneic immunocompetent C3H mice and immunocompromised nude mice models. Tumor growth was observed only in the immunocompromised models, while the immunocompetent C3H mice displayed no tumor growth. Re‐challenging the C3H mice with parental tumor cells also showed no tumor growth, but revealed an increased population of CD3^+^/CD8^+^ cells and elevated Th1 cytokine levels, suggesting systemic immunity resulting from IL‐24 treatment (Miyahara et al. 2006). In another recent study, greater levels of IFN‐γ expressing CD8^+^ cells were identified in B16 melanoma syngeneic models in response to IL‐24. An increase in the PD‐L1^+^CD45^−^ population, reflective of immune evasion, was also noted in the surviving fraction of tumor cells (Pradhan et al. 2022). The clinical trial results finally revealed that IL‐24 exhibits pro‐immune properties, stimulating the host immune response and effectively inhibiting tumor growth across various cancer types. This anti‐tumor response elicited by IL‐24 administration largely involved the activation and infiltration of CD8^+^ T cells accompanied by the release of pro‐inflammatory cytokines such as TNF, IL‐1, IFN‐γ, and IL‐12, although the precise mechanism behind these effects remains largely unknown (Tong et al. 2005). In summary, these findings indicate that IL‐24 treatment has a significant effect on the T cell population to potentially result in tumor cell killing. While information summarized above shows a significant association between IL‐24 and T cell‐mediated immune responses in cancer, the underlying molecular mechanisms and effect of IL‐24 on other immune cell subsets such as dendritic cells macrophages, and natural killer (NK) cells are not yet clear. More in‐depth studies are necessary to acquire a deeper comprehension of IL‐24's role in regulating the immune response. This knowledge could facilitate the development of combinatorial approaches using IL‐24 with immune checkpoint inhibitors for effective immunotherapy and improved treatment outcomes.

#### 2.7.7 IL‐24 Decreases Tumor Angiogenesis

Angiogenesis is a critical process responsible for the development of new blood vessels, which in turn supports tissues with vital nutrients to meet their metabolic requirements and helps maintain normal physiologic conditions. In cancer, angiogenesis is pivotal to disease progression and the development of metastases (Papetti and Herman 2002). Given that abnormal angiogenesis is a prerequisite for cancer development, a wide variety of anti‐angiogenic drugs have been approved and are currently employed clinically. However, it is important to note that a singular approach of pharmacological inhibition of angiogenesis has not proven effective in curbing tumor growth and metastasis (Lopes‐Coelho et al. 2021). Consequently, there is an increasing need to identify novel therapeutic targets able to target cancer cells at various cancer signaling pathways, including angiogenesis, in combination with existing anti‐angiogenic drugs to enhance their efficacy (Ansari et al. 2022). To this end, Saeki et al. explored the possible role of IL‐24 as an inhibitor of angiogenesis using in vitro models with human umbilical vein endothelial cells (HUVEC). Their findings illustrated that HUVECs overexpressing IL‐24 lacked tube formation capacity compared with the control. Moreover, overexpression of IL‐24 in subcutaneous lung tumor xenograft models led to a reduction in CD31 expression, supporting the hypothesis that IL‐24 overexpression inhibits in vitro differentiation of endothelial cells, potentially contributing to a decrease in tumor angiogenesis. However, this study did not elucidate the underlying mechanism responsible for this effect (Saeki et al. 2002). Subsequently, it was conclusively shown that IL‐24 inhibits tumor angiogenesis through both intracellular and extracellular mechanisms. The extracellular or secreted form of IL‐24 impeded the differentiation and migration of HUVECs and human microvascular endothelial cells (HMVEC) in a dose‐dependent manner, exhibiting stronger effects than either control treatment, that is, IFN‐γ or IP‐10. Ramesh et al. further identified that IL‐24, by binding to its receptor (IL‐22), inhibited VEGF‐mediated angiogenesis in vivo in a matrigel plug model. In a subcutaneous xenograft tumor model, where a 1:1 ratio of human lung A549 cells with parental HEK293 cells or HEK293 cells expressing IL‐24 was employed, the results indicated a reduction in tumor growth, reduced CD31 positivity as observed in immunostaining, and significantly lower hemoglobin levels in tumors containing HEK293 IL‐24, further corroborating the reduction in angiogenesis upon IL‐24 treatment (Ramesh et al. 2003). Additionally, using LNCaP and DU145 cells as a model system, Inoue et al. assessed the ability of overexpressed IL‐24 to decrease VEGF. They attributed the observed reduction in VEGF production to IL‐24 inhibiting c‐Src kinase activity, thereby abolishing STAT3 binding to the VEGF promoter. This data was further supported by findings that the IL‐24‐mediated VEGF decrease occurs in Src^+/+^ mouse embryo fibroblasts but not in Src^−/−^. The decrease in VEGF production inhibited VEGFR signaling and subsequently curtailed endothelial cell proliferation (Inoue et al. 2005). Lastly, in melanoma models, administration of IL‐24 was shown to reduce the expression of inducible nitric oxide synthase (iNOS), suggesting a negative correlation between IL‐24 and iNOS (Ekmekcioglu et al. 2003). Given that iNOS is implicated in angiogenesis, it is possible that IL‐24‐mediated anti‐angiogenic activity could also be a consequence of iNOS inhibition, a possibility worth exploring in the future. Study results summarized above reveal the promising potential of IL‐24 as an inhibitor of angiogenesis. To further validate these findings, IL‐24 has been used in combination with the angiogenesis inhibitor bevacizumab and radiation therapy, details of which will be discussed in subsequent sections of this review.

## 3 IL‐24—A Promising Anti‐Cancer Therapeutic

The summarized studies provide clear evidence that IL‐24‐mediated anti‐tumor activity operates through key pathways governing cancer progression and has undergone clinical testing for cancer treatment. Building on these initial reports, multiple laboratories, including our own, have investigated IL‐24 as a potential cancer treatment for various human cancer models, both in vitro and in vivo. Additionally, combined treatments involving radiation, chemotherapy, and small molecule inhibitors have been examined to enhance the anti‐tumor activity of IL‐24. These study results, drawn from a variety of preclinical and clinical studies, are outlined in the following sections:

### 3.1 Preclinical Studies

#### 3.1.1 IL‐24 Combinatorial Therapy With Other Drugs

Several studies indicate that a synergistic approach combining targeted therapy with conventional anti‐cancer drugs can significantly enhance overall treatment efficacy when compared with monotherapy. Consequently, research efforts have focused on combining IL‐24 targeted therapy with conventional drugs, specifically chemotherapeutic agents, to achieve substantial therapeutic outcomes. For example, Li et al. combined cisplatin treatment with an IL‐24 expressing plasmid and noted a greater reduction in mean tumor weight in a cervical cancer xenograft model compared with the plasmid control‐treated group. Their findings revealed that this combination therapy led to a greater expression of the tumor suppressor gene, nm23H1, contributing to the observed effects (Li et al. 2011). Following this, Wang et al. reported that the identical combination therapy suppressed angiogenesis and lymph‐angiogenesis in a cervical cancer xenograft model (Wang et al. 2015). IL‐24, when used in combination with 5′‐fluorouracil and doxorubicin, increased the sensitivity of colon cancer cells to both drugs in in vitro model systems (Xu et al. 2013). Combinatorial therapy with sulindac, a non‐steroidal anti‐inflammatory drug (Chandra et al. 2017; Yin et al. 2016) and adenovirus expressing IL‐24 was assessed in in vitro and in vivo NSCLC models and demonstrated enhanced growth inhibition compared with monotherapy. Use of sulindac increased the half‐life of IL‐24 and increased the expression of various protein kinases and caspases, ultimately leading to greater apoptosis of tumor cells (Oida et al. 2005). Another recent study revealed that IL‐24 administration increased the expression of the epithelial marker E‐Cadherin and suppressed Zeb1 in glioblastoma multiforme (GBM) cell lines, leading to increased sensitivity to temozolomide. Additionally, administration of IL‐24 led to decreased Zeb1 protein stability and transcription in GBM cells (Lin et al. 2021).

#### 3.1.2 IL‐24 and Small Molecule Inhibitors

**FIGURE 3:**
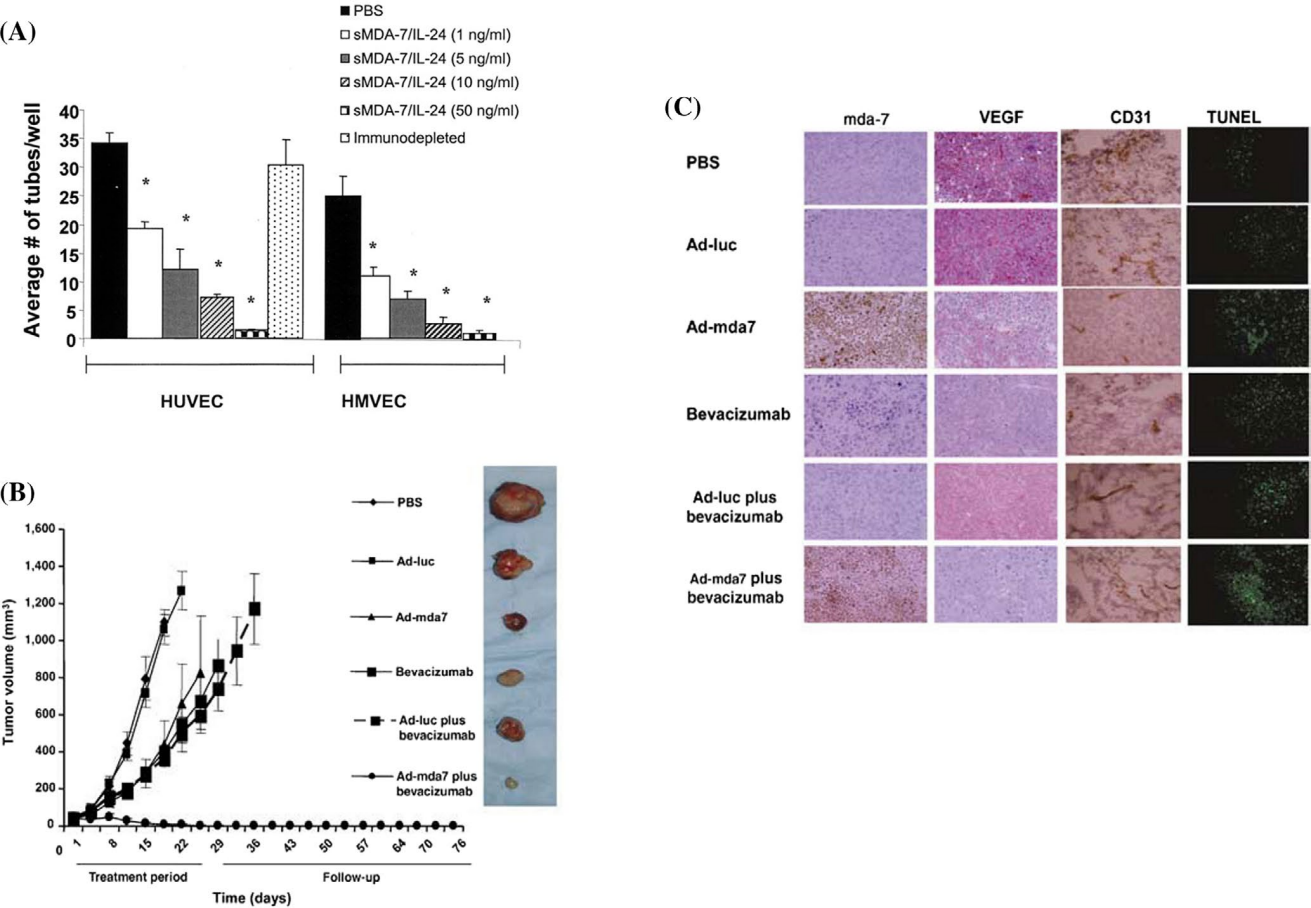
Effect of IL‐24 on tumor angiogenesis. Adenovirus‐based delivery of IL‐24 retards (A) tube formation abilities of HUVEC and HMVEC cells in vitro. Additional in vivo experiments on subcutaneous human lung cancer xenografts showed (B) combinatorial therapy involving adenovirus‐mediated IL‐24 (Ad‐mda7) delivery with bevacizumab effectively controlled tumor growth. Immunohistochemical analysis further revealed (C) decreased VEGF and CD31 expression along with marked increase in TUNEL staining in the Ad‐mda7 + bevacizumab group compared with controls. Images reproduced from Ramesh et al. (2003) and Inoue et al. (2007) that is under open access Creative Commons CC BY 4.0 license. Image was generated with Biorender.com.

Small‐molecule inhibitors are targeted drugs that are currently employed in cancer therapy. While they offer advantages such as enhanced pharmacokinetics, and the ability to bind to both intracellular and extracellular targets, they present with certain challenges, including drug resistance with prolonged use and limited effectiveness in specific groups of patients. To address these challenges, a combination therapy approach is being adopted, where small molecule inhibitors are combined with other targeted therapies (Zhong et al. 2021). One such strategy involves combining small molecule inhibitors with IL‐24. Given the promising preclinical data showcasing IL‐24's potential as an angiogenesis inhibitor, researchers have explored the therapeutic potential of IL‐24 in combination with bevacizumab, a recognized angiogenesis inhibitor, in NSCLC models. Interestingly, Inoue et al., determined that conditioned medium from H1299 cells treated with adenovirus expressing IL‐24 and bevacizumab, induced apoptosis and of HUVECs. They also showed that the same treatment combination completely inhibited tumor growth in subcutaneous lung tumor xenograft model with no observed side effects or toxicity; the combination delayed tumor growth by 76 days compared with only 8 days with monotherapy (Inoue et al. 2007) (Figure 3).

In another study, synergistic activity between geldanamycin (GA), an Hsp90 inhibitor, and IL‐24 was shown in pancreatic cancer cells, although the underlying mechanism contributing to this effect remains unclear (Zhang et al. 2013). Favorable therapeutic outcomes were also reported for the combination of IL‐24 with Trastuzumab (Herceptin) (McKenzie et al. 2004), as well as HDAC inhibitors (Hamed, Das, et al. 2013; Hamed, Yacoub, et al. 2013). Further details of these combinations are summarized in Table 2.

**TABLE 2:** Current IL‐24 based therapies for cancer.

Delivery system	Therapy/drug used	Cancer type	In vitro model	In vitro outcome	In vivo model	In vivo outcome	References
Adenovirus (AdBB‐IL‐24)	5‐fluorouracil and Doxorubicin	Colon cancer	HT‐29; SW480; SW620; L‐02; MRC‐5	Inhibition of colon cancer cell growth; Tumor specific apoptosis	Xenograft tumor model	Confirmation of enhanced anti‐tumor effects	Xu et al. (2013)
Adenovirus (ZD55‐IL‐24)	Dacarbazine	Melanoma	A375; M14	Increased in melanoma cell apoptosis; Enhanced anti‐tumor activity	NA	NA	Jiang et al. (2010)
Adenovirus (Ad.5/3‐mda‐7)	BI‐69A11 (inhibitor)	Colon cancer	HT‐29; HCT15; HCT116; SW480	Inhibits cell invasion and angiogenesis; Inhibition of cell proliferation	Xenograft tumor model	Inhibits tumor growth; anti‐angiogenic activity; decreased tumor volume; increased apoptosis	Pal et al. (2014)
Adenovirus (Ad‐mda‐7)	5‐flurouracil; cisplatin; mitomycin C; etoposide	Esophageal carcinoma	TE‐1; TE‐2; TE‐10; TE‐11 YES‐2; YES‐4; YES‐5; YES‐6; A549; Human Fibroblasts; OUMS‐24; HFF	Increased cell arrest (IL‐24 + 5FU); induced p53 phosphorylation and extracellular protein kinases 1 and 2 (IL‐24 + 5FU); Ccmbined cytotoxic effects	NA	NA	Ma et al. (2014)
Plasmid (pDC316‐hIL‐24)	Cisplatin	Cervical cancer	NA	NA	Xenograft tumor model	Reduced tumor growth; decreased metastasis of tumor lymph node; increased lymphangiogenesis	Wang et al. (2015)
Oncolytic Adenovirus (ZD55‐IL‐24)	Ionizing radiation	Prostate cancer	PC‐3; DU‐145; HEK‐293	Reduced cell proliferation; increased expression of apoptosis proteins	Xenograft tumor model	Reduced volume of xenograft tumor; increased rate of apoptosis	Mao et al. (2020)
Adenovirus (Ad.5.mda‐7/Ad.5/3‐mda‐7)	BI‐97C1	Colon cancer	HCT116; RKO colorectal carcinoma	Inhibits growth of RKO cells both in vitro and in vivo; enhanced cytotoxicity; enhanced cell death	Xenograft tumor model	Reduced tumor growth; enhanced signs of anti‐angiogenesis; reduced cell proliferation	Azab et al. (2012)
Adenovirus (Ad‐mda7)	Bevacizumab	Lung cancer	Umbilical vein endothelial cells	Inhibits endothelial cell proliferation; Shows antiangiogenic effects	Xenograft tumor model	Anti‐angiogenic activity; enhanced rate of tumor inhibition	Inoue et al. (2007)
Adenovirus (type 5 Ad‐mda‐7)	Heat‐shock protein 90 inhibitor (hsp 90 inhibitor)	Pancreatic cancer	PANC‐1; AsC‐1; MIA‐PaCa‐2; BxPC‐3; human keratinocytes; HaCaT	Greater cytotoxic effects; tumor growth suppression; increased G1 cell cycle arrest	NA	NA	Zhang et al. (2013)
Adenovirus (Ad‐mda‐7)	Ionizing Radiation	Lung cancer	A549	Decreased angiogenic effects from radiation (measured in bFGF and VEGF expression); decreased microvessel density; Increased cell apoptosis	Xenograft tumor model	Increased apoptosis; long lasting tumor growth delay; enhanced apoptosis in tumor cells	Nishikawa et al. (2004)
Shuttle Vector/Plasmid (pDC316‐hIL‐24)	Cisplatin	Cervical cancer	NA	NA	Hela cell xenograft tumor model	Decreased mean tumor growth; showed less decrease in body weight	Li et al. (2011)
Oncolytic Adenovirus (ZD55‐IL‐24)	TRAIL adenovirus	Colorectal cancer	HEK293; SW620; HCT116; HT29; Hep3B; A549; NHLF; MRC5	In vitro inhibition of cell proliferation; enhanced additive induction of apoptosis	Xenograft tumor model	Reduced tumor growth in tumor; increased tumor cell apoptosis	Zhao et al. (2006)
Oncolytic Adenovirus (AD‐enAFP‐IL‐24)	Suppressor of Cytokine Signaling 3 Gene (SOCS3 gene)	Liver cancer	HepG2; Hep3B; MRC5; HuH7; PLC/PRF/5; HEK293	Increased targeting of tumor cells (tumoricidal); increased tumor cell apoptosis	Xenograft tumor model	Showed most “potent” inhibition of tumor growth in vivo	Cao et al. (2011)
Adenovirus (Ad‐Rz/IL‐24)	Anti‐VEGF ribozyme adenovirus	Colon cancer	HT‐29; xenograft tumor model	Downregulation of VEGF mRNA expression; decreased cell viability and growth inhibition; enhanced cancer cell apoptosis	Xenograft tumor model	Antiangiogenic effects on tumor	Chang et al. (2009)
Oncolytic Adenovirus (ZD55‐IL‐24)	Immune Checkpoint Blockade [PD‐1 Blockade]	Melanoma	HEK293; B16	Enhanced infiltration of immune response in tumor; increase in regulatory T cells	B‐16‐bearing immunocompetent mouse model	Decreased tumor establishment/weight/volume; increased tumor infiltration; improved antitumor response for distant tumors	Hu et al. (2021)
Adenovirus (Ad‐IL‐24‐OSM)	OSM Adenovirus	Melanoma	BJ5183; QBI‐293A; A375; xenograft tumor model	Increased cell growth suppression; increased cell apoptosis; enhanced G1 cell cycle arrest; increased antiangiogenic activity	Xenograft tumor model	Decreased tumor growth; enhanced tumor suppression	Xu et al. (2014)
Adenovirus (Ad.5/3‐mda‐7)	BI‐97C1 (Sabutoclax)	Prostate cancer	Human prostate cells; xenograft tumor model; spontaneous immunocompetent transgenic mouse model	Increased translocation of mitochondrial Bax; increased cancer cell apoptosis; slight increase in the autophagy response	Spontaneous immunocompetent transgenic mouse model	Reduced tumor growth; reduced cell volume	Dash et al. (2011)
Adenovirus (Ad.5/3‐PEG‐E1A‐mda‐7)	Histone Deacetylase Inhibitors (HDACIs)	Renal carcinoma	Renal carcinoma cells	Enhanced additive cell killing	Murine model	Suppression of tumor growth; enhanced suppression of bystander tumor growth	Hamed, Das, et al. (2013)
DNA Vaccine (pcDNA3.1‐MDA/IL‐24)	E7 DNA Vaccine	Cervical cancer	EL4	Increased cytotoxicity	Mouse model	Increased cytotoxic activity in the cells; increased cytokine recruitment; increased IL‐24 infiltration; significant decrease in tumor volume	Miri et al. (2022)
Oncolytic Vaccinia Virus (VV‐IL‐24)	Luteolin	Liver cancer	LO2; MHCC97‐H; HepG2; PLC/PRF/5, Hep3B; HEK293	Decreased cell viability; enhanced synergistic effects on liver cells; higher amounts of apoptosis	Xenograft tumor model	Inhibit tumor progression	Wang, Li, et al. (2021)
IL24‐iRGD	iRGD tumor‐specific peptide	Prostate cancer	PC‐3; RWPE‐1	Increased cell apoptosis; inhibition of tumor cell growth	Non‐specified tumor model	Enhanced binding and penetration to tumor tissue in vivo; reduced tumor growth; increased tumor cell apoptosis	Yang et al. (2019)
Adenovirus (pZD55‐IL‐24)	Temozolomide	Melanoma	A375; MV3; Human Lung Fibroblasts	Enhanced infectivity into tumor cells; increased levels of apoptosis	Non‐specific tumor model	Significant inhibition of tumor growth; reduction in total tumor volume	Yang et al. (2018)
Oncolytic Adenovirus (ZD55‐IL‐24)	Paclitaxel	Breast cancer	MDA‐MB‐231; Bcap‐37; HEK‐293	Increased cytotoxicity; increased uptake of combined therapeutic; increased apoptosis	NA	NA	Fang et al. (2013)
Adenovirus (ONCO) (ZD55‐IL‐24)	Radiotherapy	Melanoma	A375	Inhibition of cell proliferation; increased cell apoptosis	Xenograft tumor model	Decreased tumor size; enhanced tumor cell apoptosis	Jiang et al. (2012)
Adenovirus (Ad‐mda‐7)	Transtuzumab	Breast cancer	SKBr3; MCF‐7; MCF‐7‐Her‐18	Increased cytotoxicity and tumor cell death	Xenograft Tumor Model	Tumor growth inhibition	McKenzie et al. (2004)
Adenovirus (Ad‐mda‐7)	Radiotherapy	Breast cancer	T47D; MCF‐7; MDA‐MB‐453; SKBr3; MDA‐MB‐231, MDA‐MB‐468; MDA‐MB‐361; HBL‐100; BT‐20	Reduced cell proliferation; reduced cell growth; enhanced cell apoptosis	Xenograft tumor model	Reduced tumor size; enhanced cell death	Chada et al. (2006)
Adenovirus (Ad‐mda‐7)	Ionizing radiation	Glioma	Fischer 344 rat RT2 glioma; primary rodent astrocytes	Enhanced cell death; increased apoptosis; reduced mitochondrial free radical generation causing anti‐proliferation effects	Fischer 344 rats	Enhanced survival rate	Yacoub, Mitchell, Lister, et al. (2003)
Adenovirus serotype 5/3 (Ad5/3.PEG‐E1.mda‐7)	Histone Deacetylase Inhibitors (HDACIs)	Glioblastoma	A549; primary human GBM	Enhanced toxicity in vitro; increased levels of autophagy in vitro	Xenograft tumor model	Increased survival in vivo; increased tumor growth suppression; selective tumor cell death	Hamed, Yacoub, et al. (2013)
Adenovirus (Ad‐mda‐7)	Vitamin E succinate	Ovarian cancer	MDA H2774	Enhanced anti‐tumor effect	NA	NA	Shanker et al. (2007)
Adenovirus (Ad‐mda‐7)	Perillyl alcohol	Pancreatic cancer	AsPC‐1; MIA PaCa‐2; PANC‐1; BxPC‐3	Induces ROS and reverses the mda‐7/IL‐24 “protein translational block”; tumor growth suppression and increased apoptosis	NA	NA	Lebedeva et al. (2008)

#### 3.1.3 IL‐24 and Radiation Therapy

Radiation therapy is critical to cancer treatment and is known to generate free radicals leading to DNA damage and triggering cell death. Radiation is often combined with other therapeutic modalities such as surgery, chemotherapy, or immunotherapy. While ionizing radiation continues to stand out as one of the most effective choices for cancer therapy, it has some limitations, particularly in terms of its impact on normal cells. Recognizing these limitations, radiation therapy is now being utilized in combination with targeted therapies to enhance the overall therapeutic outcome. In this context, several studies employing various in vitro and in vivo models have demonstrated that IL‐24 treatment sensitizes cancer cells to radiation therapy. For instance, Nishikawa et al. evaluated the efficacy of IL‐24 administered in combination with ionizing radiation for the management of NSCLC using A549 xenograft tumors in nude mice models. The combination resulted in reduced levels of VEGF, basic fibroblast growth factor (bFGF), and micro vessel density and increased tumor cell apoptosis compared with individual monotherapy. Exogenous IL‐24 also demonstrated the ability to sensitize endothelial cells to ionizing radiation (Nishikawa et al. 2004). Similar approaches assessing the combinatorial effect of IL‐24 and radiation therapy have been employed in in vivo xenograft models of melanoma (Jiang et al. 2012), glioma (Yacoub, Mitchell, Lister, et al. 2003) and breast cancer (Chada et al. 2006). Additionally, synergistic anti‐tumor effects have been observed upon using ZD55‐IL‐24, an oncolytic adenovirus containing IL‐24, in combination with radiation in prostate cancer models (Mao et al. 2020). The results from these in vivo studies consistently showed increased tumor cell apoptosis and a notable decrease in tumor size, thereby providing a strong rationale for utilizing IL‐24 in combination with radiation therapy to improve cancer treatment outcomes.

#### 3.1.4 IL‐24 and Immune Checkpoint Inhibitors

The combination of IL‐24 and immune checkpoint inhibitors has recently gained importance in the field of cancer treatment. For instance, Hu et al. conducted a study involving localized ZD‐55‐IL24 administration along with systemic PD‐1 blockade in an immunocompetent mouse model bearing B16 melanoma. The combination demonstrated a synergistic therapeutic effect, characterized by tumor growth inhibition compared with either monotherapy, as well as a notable increase in tumor immune cell infiltration, affecting local as well as distant tumors (Hu et al. 2021). Similar anti‐tumor benefits were reported in a study combining a therapeutic DNA vaccine with IL‐24 and IL‐10 blockade in models of HPV 16^+^ cervical cancer (Miri et al. 2022). More recently, Fisher and colleagues utilized a genetic engineering approach to modify IL‐24 and create a designer cytokine with enhanced secretion and stability that they termed M7S (IL‐24S). Adenovirus expressing M7S led to greater cellular apoptosis and tumor suppression compared with wild type IL‐24; enhanced anti‐tumor and bystander effects were observed in melanoma and prostate cancer xenograft models. Furthermore, M7S in combination with immune checkpoint inhibitors, specifically anti‐PD‐L1, led to more robust tumor suppression in syngeneic melanoma models than the combination with IL‐24 (Pradhan et al. 2022). As a result, combination therapy of IL‐24 with immune checkpoint inhibitors is considered a promising strategy to improve therapeutic outcomes in cancer.

#### 3.1.5 Oncolytic Virus‐Based IL‐24 Therapy

Oncolytic virus‐based IL‐24 therapy has also gained prominence in recent years. Deng et al. assessed the anti‐tumor efficacy of an IL‐24 delivered using vaccinia virus (Guang9 strain harboring IL‐24; VG9‐IL‐24) and showed dose‐dependent cytotoxicity in colorectal cancer cell lines. Additionally, VG9‐IL‐24 treatment caused G2/M phase cell cycle arrest and induced apoptosis. These effects of VG9‐IL‐24 were mediated by increasing PKR, JNK, and decreasing STAT3 phosphorylation. In vivo studies showed treatment of HCT116 colorectal tumor‐bearing mice with VG9‐IL‐24 enhanced survival rates compared with control groups (Deng et al. 2021). VG9‐IL‐24 treatment also delayed MDA‐MB‐231 breast cancer tumor growth and proved effective in delaying tumor growth in xenograft mouse models using the cell line in vivo (Deng et al. 2020). Likewise, the efficacy of ZD55‐IL‐24, an oncolytic adenovirus for IL‐24 delivery, in combination with the chemotherapeutic drug paclitaxel was examined in breast cancer models. This combination increased cell apoptosis and led to greater cytotoxicity in MDA‐MB‐231 and Bcap‐37 cells (Fang et al. 2013). Furthermore, Hu et al. investigated the efficacy and the associated mechanism by which ZD55‐IL‐24 inhibited growth of human A375 and mouse B16 melanoma cells. They identified different mechanisms of cell killing in the two models studied. In the immunocompetent model, ZD55‐IL‐24 remodeled the tumor microenvironment and contributed to the expression of cytokines involved in systemic anti‐tumor immunity. Additionally, ZD55‐IL‐24 shifted the tumor cells from a “self” state to a “non‐self” state, further inhibiting angiogenesis. This, in turn, contributed to tumor growth inhibition and enhanced survival in mice. In contrast, ZD55‐IL‐24 exerted its effect via a direct cell‐killing pathway in the immunocompromised models (Hu et al. 2020).

#### 3.1.6 Mesenchymal Stem Cell‐Based IL‐24 Therapy

Apart from their pronounced role in tissue repair and regeneration, mesenchymal stem cells (MSCs) have garnered attention as attractive carriers for delivering anti‐cancer payloads. A comprehensive review of MSCs as a choice of drug delivery vehicle for cancer therapeutics can be found in an article by Takayama et al. (2021). Fan et al. investigated the utility of umbilical cord‐derived MSCs (UC‐MSCs) as a delivery vehicle for IL‐24. They utilized IL‐24 overexpressing UC‐MSCs (IL‐24‐UC‐MSCs), which reduced tumor cell proliferation by increasing apoptosis in U251 glioma models in vitro. Similarly, tail vein injection of IL‐24‐UC‐MSCs resulted in suppression of tumor growth in glioma xenograft models compared with control groups (Fan et al. 2020). In a study conducted by Wu et al., the anti‐tumor properties of MSCs derived from induced pluripotent stem cells (iPSCs) expressing IL‐24 were explored. These IL‐24 integrated MSCs (IL‐24‐iMSCs) were co‐cultured with B16‐F10 mouse melanoma cells and induced apoptosis. Data from in vivo studies indicated that retro‐orbital injection of IL‐24‐iMSCs effectively inhibited the growth of melanoma tumors in subcutaneous mouse models (Wu et al. 2020). These studies highlight a promising role for MSC‐based delivery of IL‐24 to tumors in enhancing therapeutic outcomes. MSC‐based gene delivery offers advantages regarding ease of isolation and therapeutic safety and efficacy and is widely employed in clinical trials for cancer treatment. However, additional research is required to identify potential long‐term risks associated with MSC therapy for gene delivery before its translation to the clinical setting.

#### 3.1.7 Other Approaches

In addition to the aforementioned strategies, a few studies have examined the synergistic effects of IL‐24 with TRAIL adenovirus (Zhao et al. 2006), anti‐VEGF ribozyme adenovirus (Chang et al. 2009), and OSM adenovirus (Xu et al. 2014) in various in vitro and in vivo cancer models, demonstrating promising therapeutic outcomes.

**FIGURE 4:**
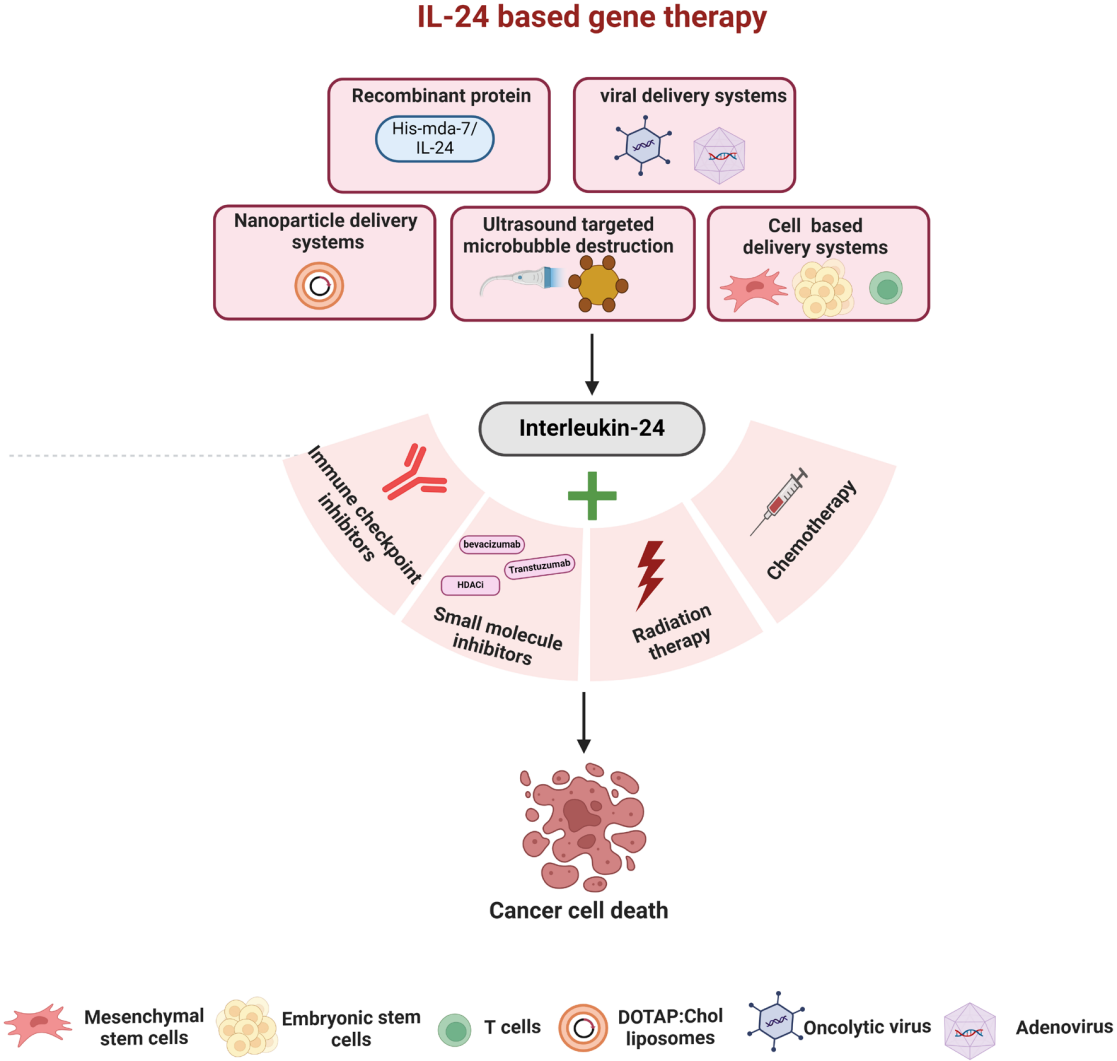
Schematic representation of IL‐24 drug delivery systems and combinatorial therapy approaches currently used in the cancer setting. Image created with biorender.com.

In summary, the currently employed combinatorial therapy in cancer research has undeniably demonstrated efficacy in producing potent anti‐cancer effects in various preclinical models and clinical trials. The notable effectiveness of IL‐24‐based combination therapy regimens stems from the ability of combined therapies to concurrently target multiple key pathways contributing to tumor heterogeneity, thus countering resistance that may arise from individual therapies. The previously mentioned studies illustrate the effectiveness of IL‐24 in combination with chemotherapeutic drugs, small molecule inhibitors, radiation therapy, and other approaches (Figure 4) in modulating key pathways and restraining tumor growth across diverse preclinical cancer models. However, these combination therapies require extensive testing in clinical trials to gain a more comprehensive understanding of their toxicity profiles and modulation of treatment outcomes.

## 4 Clinical Trials

Compelling evidence from a multitude of preclinical models (in vitro and in vivo) regarding the use of IL‐24 as an anti‐cancer therapeutic has paved its way into clinical trials. In 2005, a Phase‐I clinical trial involving intra‐tumoral administration of INGN 241, an adenovirus construct expressing the IL‐24 transgene, was conducted in 28 patients with resectable solid tumors representing 15 different tumor types, who had received prior treatment with surgery in combination with radiation or chemotherapy. The treatment employed a dose escalation approach and administered 2 × 10^10^ to 2 × 10^12^ viral particles directed toward the central region of the tumor. The administration of INGN 241 resulted in the localization of IL‐24 in tumor cells, inducing tumor cell apoptosis, as indicated by the TUNEL assay. Additionally, profound bioactivity was observed, with induction of IL‐24 protein and no significant toxicity. This clinical trial established INGN‐241 therapy to be well tolerated in patients and underscored its immense clinical potential (Cunningham et al. 2005). Subsequently, Tong et al. assessed the efficacy of intra‐tumoral administration of INGN 241 in 22 advanced cancer patients. INGN 241 treatment exhibited a pro‐apoptotic activity, characterized by decreased Ki‐67 staining. Furthermore, like preclinical models, intra‐tumoral INGN 241 treatment modulated the expression of key downstream genes such as β‐catenin and iNOS in treated patients. Increased serum cytokine levels of IL‐10, IL‐6, and TNF‐α were indicative of an activated systemic immune response in patients who received INGN 241. Notably, treatment with INGN 241, at day 15, led to a robust increase in CD3^+^ and CD8^+^ T cell populations, predicting a Th1 response (Tong et al. 2005). The outcomes of these studies are highly encouraging and serve as evidence for the successful translation of bench to bedside concepts employed in the realm of IL‐24 as a cancer therapeutic.

## 5 IL‐24 Based Therapy in the Clinic: The Challenge and the Way Forward

Despite numerous preclinical studies demonstrating the selective anti‐tumorigenic potential of IL‐24 in tumor cells, there has been limited progress in the last 20 years on moving IL‐24 into clinical trials. Several potential hurdles in the successful translation of IL‐24‐based therapy into clinical practice are outlined below:

- While secreted IL‐24 has robust anti‐tumor and anti‐angiogenic effects, there is limited exploration in this area. Additionally, research conducted in our laboratory revealed technical challenges related to large‐scale purification of IL‐24, which hinders progress. Thus, developing improved strategies to overcome these technical difficulties would significantly advance IL‐24‐based therapy for cancer.
- The vast majority of IL‐24 based research findings from academia have not transitioned into clinical trials, largely due to funding and regulatory issues.
- Genetic approaches to enhance IL‐24 stability and secretion could potentially amplify its anti‐tumor effects.
- The role of lncRNAs in regulating IL‐24 is an area that warrants in‐depth exploration.
- Cell‐based delivery systems for delivery of IL‐24 are gaining importance. While a vast majority of studies utilize MSC‐based therapy for IL‐24 delivery due to their lower immunogenicity compared with other delivery systems, the use of other cell‐based delivery systems for IL‐24 delivery represents a novel approach.

While there is preliminary evidence regarding the efficacy of IL‐24 in other diseases, it has not been comprehensively investigated. Further research focused on elucidating the biology and role of IL‐24 in CVD, eye diseases, infections, and allergies could help bridge the existing gaps and identify new treatment strategies. In summary, the multifunctional cytokine IL‐24 has gained clinical relevance in the field of cancer. Additionally, results from various preclinical studies suggest the enhanced efficacy of IL‐24 in combination with other treatment modalities, which warrants further exploration in a clinical setting to evaluate toxicity, immune response, and ultimately translate these findings into clinical practice. These combination approaches have the potential to overcome acquired resistance to conventional therapies and improve patient responses to treatment. Nevertheless, the potential correlation between IL‐24 and other human diseases is slowly emerging and necessitates thorough investigations. Understanding the complex regulatory functions of IL‐24 biology in other human diseases holds the promise of developing novel therapeutic strategies for managing both these conditions and cancer.

## Author Contributions

**Rajeswari Raguraman:** conceptualization (lead), data curation (lead), writing – original draft (lead), writing – review and editing (equal). **Anupama Munshi:** conceptualization (equal), funding acquisition (supporting), investigation (supporting), writing – review and editing (equal). **Rajagopal Ramesh:** conceptualization (supporting), funding acquisition (lead), project administration (lead), resources (lead), writing – original draft (supporting), writing – review and editing (equal).

## Conflicts of Interest

The authors declare no conflicts of interest.

## Related WIREs Articles

The importance of the IL‐1 family of cytokines in nanoimmunosafety and nanotoxicology

## Data Availability

Data sharing is not application to this article as no new data were created or analyzed in the study.
